# Systematic review and meta-analysis of myocarditis and pericarditis in adolescents following COVID-19 BNT162b2 vaccination

**DOI:** 10.1038/s41541-023-00681-3

**Published:** 2023-06-09

**Authors:** Patrick D. M. C. Katoto, Liliane N. Byamungu, Amanda S. Brand, Jacques L. Tamuzi, Mireille A. M. Kakubu, Charles S. Wiysonge, Glenda Gray

**Affiliations:** 1 Office of the President and CEO, South African Medical Research Council, Cape Town, South Africa; 2 Centre for Evidence-based Health Care, Division of Epidemiology and Biostatistics, Department of Global Health, Faculty of Medicine and Health Sciences, Stellenbosch University, Cape Town, South Africa; 3 Centre for Tropical Diseases and Global Health, Department of Medicine, Catholic University of Bukavu, Bukavu, Democratic Republic of the Congo; 4 Department of Pediatric, Faculty of Medicine and Health Sciences, University of KwaZulu-Natal, Durban, South Africa; 5 Ministry of Health and Social Services of Namibia, Windhoek, Namibia; 6 Cochrane South Africa, South African Medical Research Council, Cape Town, South Africa; 7 HIV and other Infectious Diseases Research Unit, South African Medical Research Council, Durban, South Africa

**Keywords:** Epidemiology, Medical research

## Abstract

Myocarditis and pericarditis are frequent complications of COVID-19, but have also been reported following vaccination against COVID-19 in adolescents. To build vaccine confidence and inform policy, we characterized the incidence of myocarditis/pericarditis in adolescents following BNT162b2 vaccination and explored the association with dose and sex. We searched national and international databases for studies reporting the incidence of myocarditis/pericarditis following BNT162b2 vaccination as the primary endpoint. The intra-study risk of bias was appraised, and random-effects meta-analyses were performed to estimate the pooled incidence by dose stratified by sex. The pooled incidence of myocarditis/pericarditis was 4.5 (95%CI: 3.14–6.11) per 100,000 vaccinations across all doses. Compared to dose 1, the risk was significantly higher after dose 2 (RR: 8.62, 95%CI: 5.71–13.03). However, adolescents experienced a low risk after a booster dose than after dose 2 (RR: 0.06; 95%CI: 0.04–0.09). Males were approximately seven times (RR: 6.66, 95%CI: 4.77–4.29) more likely than females to present myocarditis/pericarditis. In conclusion, we found a low frequency of myocarditis/pericarditis after BNT162b2, which occurred predominantly after the second dose in male adolescents. The prognosis appears to be favorable, with full recovery in both males and females. National programs are recommended to adopt the causality framework to reduce overreporting, which undercuts the value of the COVID-19 vaccine on adolescent life, as well as to extend the inter-dose interval policy, which has been linked to a lower frequency of myocarditis/pericarditis.

## Introduction

In postmarketing studies, myocarditis and other inflammatory heart disorders have been reported among serious adverse events following mRNA COVID-19 vaccination in adolescents^1,2^. In weighing risks and benefits the United States Advisory Committee on Immunization Practices (ACIP), for example, found that the documented hazards of COVID-19 infection and its potentially severe complication (such as hospitalization, death, and long COVID) exceeded the potential risks of experiencing rare adverse events following vaccination, including the risk of developing myocarditis and pericarditis^3^.

The stated benefit of the mRNA vaccine in adolescents will necessitate dynamic evidence. On the one hand, SARS-CoV-2 has a propensity to mutate and consequently develop new variants or even sub-variants, which will influence the evidence at a specific time. On the other hand, if myocarditis linked to the mRNA COVID-19 vaccination does not resolve or is misreported, it may lead to long-term complications in healthy adolescents and to vaccine hesitancy that extends to other vaccinations throughout the life course. As a result, low vaccination uptake due to vaccine hesitancy may undercut vaccine’s significant role in averting severe disease, post-COVID complications, and mortality. Hence, dynamic evidence is also essential for informing global policy on the COVID-19 mass immunization of adolescents^4–8^.

In fact, this difference in methodological approaches used for safety surveillance is mirrored in the interpretation of data and the variety of related policies. This variety ranges from adolescents receiving no COVID-19 vaccination at all to adolescents receiving one or two primary doses plus booster doses. Furthermore, the variation in time between SARS-CoV-2 vaccination doses may influence the incidence of inflammatory adverse reactions, as a shorter gap in dosage interval may increase the risk of myocarditis and pericarditis compared to a longer one^9,10^. This inconsistency may exacerbate the global inequity in vaccine distribution, particularly in vulnerable communities where the pandemic is harming the economic and social development of children and adolescents^11^. Hence, we aimed to consolidate the existing evidence on the incidence and risk of myocarditis and pericarditis following BNT162b2 vaccination against COVID-19 in adolescents to inform national and global policies.

## Results

### Database searching for post-vaccine myocarditis studies

**Fig. 1:**
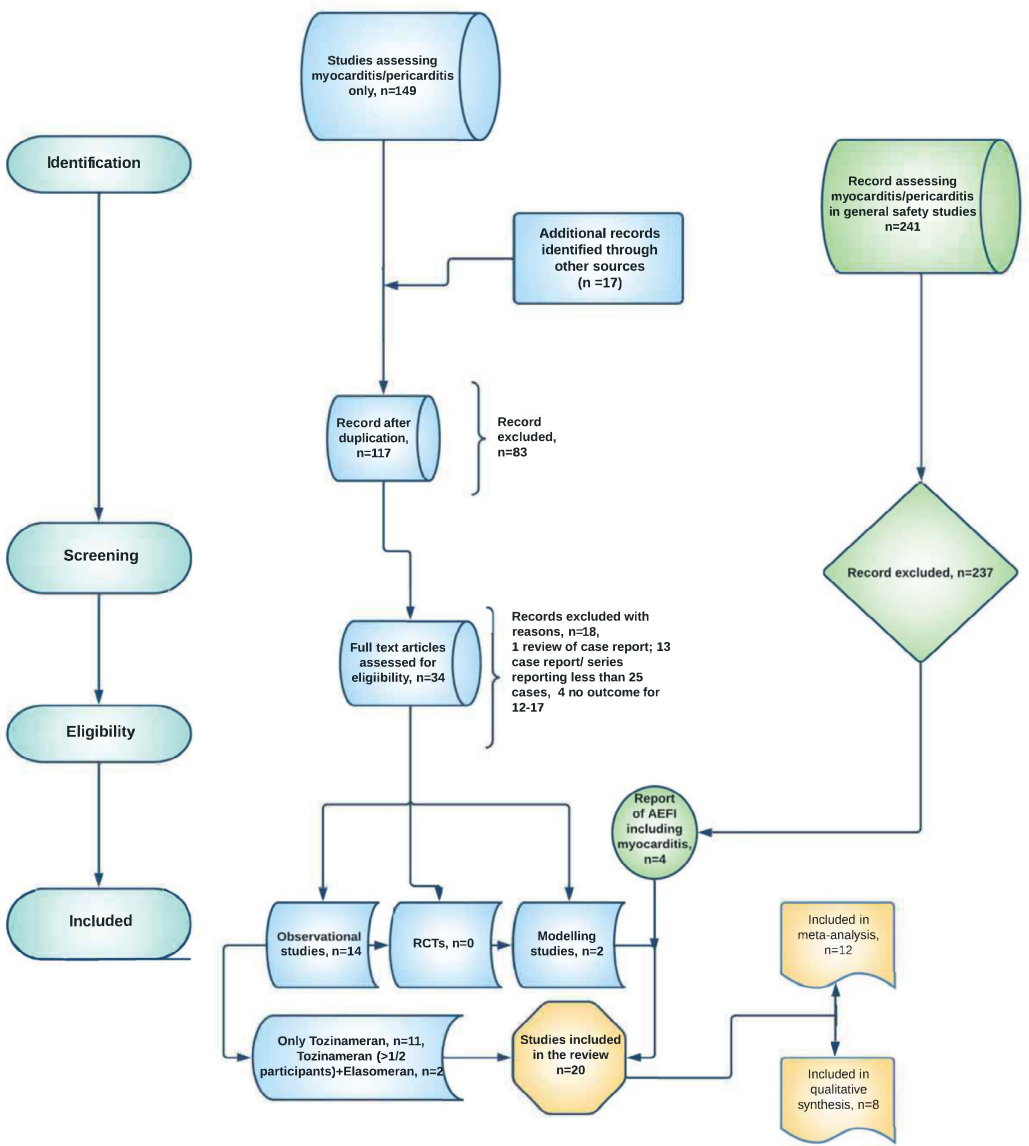
Study flow chart diagram. RCTs: Randomized controlled trials, AEFIs: Adverse events following immunization.

The database search for studies with myocarditis as a distinct outcome yielded 149 publications, with an additional 17 articles retrieved from other sources, such as reference lists of related papers. After removing 49 duplicates, 117 papers were assessed, with 83 clearly irrelevant publications excluded at the title/abstract screening stage (see ineligibility criteria in the method section). This left 34 full-text articles for consideration, with 16 matching the requirements for final inclusion. Beyond the database searches for publications with myocarditis as a distinct outcome, we also found 241 publications of safety studies that reported myocarditis as adverse events following immunization (AEFIs). Four of the general safety studies met our inclusion criteria, bringing the number of eligible studies in this review to twenty. Twelve of the twenty studies were included in the quantitative synthesis, while data from eight studies were narratively synthesized (Fig. 1). The inclusion period for participants in the 20 studies spanned the period from December 2020 to February 2022 (Supplementary Table 1). There were no studies found in the African region that met our inclusion criteria (Supplementary Fig. 1).

### Risk of bias in the included studies

The risk of bias (RoB) was evaluated in 20 studies^1,2,4,6,7,12–26^ using ROBINS-I. The assessments for RoB domains 1 to 5, which include confounding, selection bias, bias in the classification of interventions, bias due to deviations from intended interventions, and missing data bias, are summarized in Supplementary Table 2. The assessments for domains 6 to 9, which primarily address bias in the measurement of outcomes, bias in the selection of the reported result, other sources of bias, and overall bias, are presented in Supplementary Table 3.

A total of 14 studies (70%) had a high overall RoB^4,6,7,12,14,16,17,19,21,23,27^. Most studies were judged to have a high overall RoB due to a lack of adjustment for potential confounders (e.g.: age, gender, prior infection history, SARS-CoV-2 variants, comorbidities, etc.). but some presented selection bias or potential for misclassification of myocarditis. All these studies had one or more additional domain at unclear RoB. The remaining studies (*n* = 6; 30%) were all at unclear overall RoB^2,13,15,17,20,22^.

### Myocarditis or pericarditis by vaccine dose

**Fig. 2:**
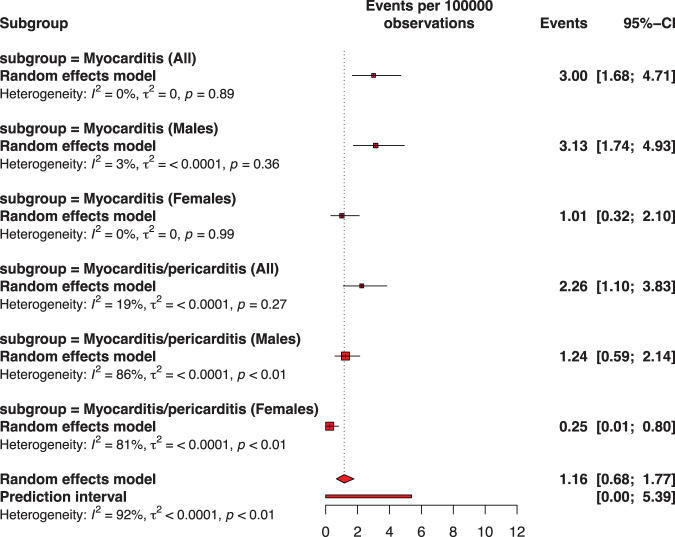
Pooled proportion of myocarditis or of myocarditis/pericarditis following the first dose of BNT162b2 vaccination against COVID-19 among adolescents. Subgroup are presented to consider sex. The pooled incidence of studied inflammatory heart conditions following the first dose was 1.16 cases per 100,000 doses of BNT162b2 vaccine. The whisker represents the 95% confidence interval. The inclusion period of participants spanned from December 2020 to February 2022.

**Fig. 3:**
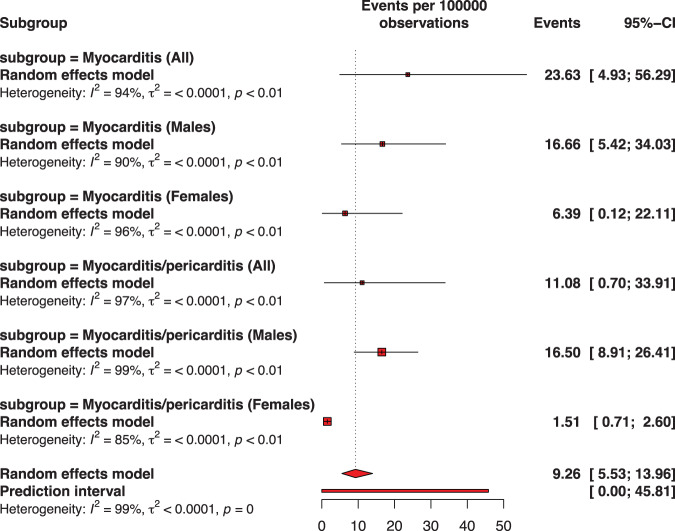
Pooled proportion of myocarditis or of myocarditis/pericarditis following the second dose of BNT162b2 vaccination against COVID-19 among adolescents. Subgroup are presented to consider sex. The pooled incidence of studied inflammatory heart conditions following the second dose was 9.26 cases per 100,000 doses of BNT162b2 vaccine. The whisker represents the 95% confidence interval. The inclusion period of participants spanned from December 2020 to February 2022.

**Fig. 4:**
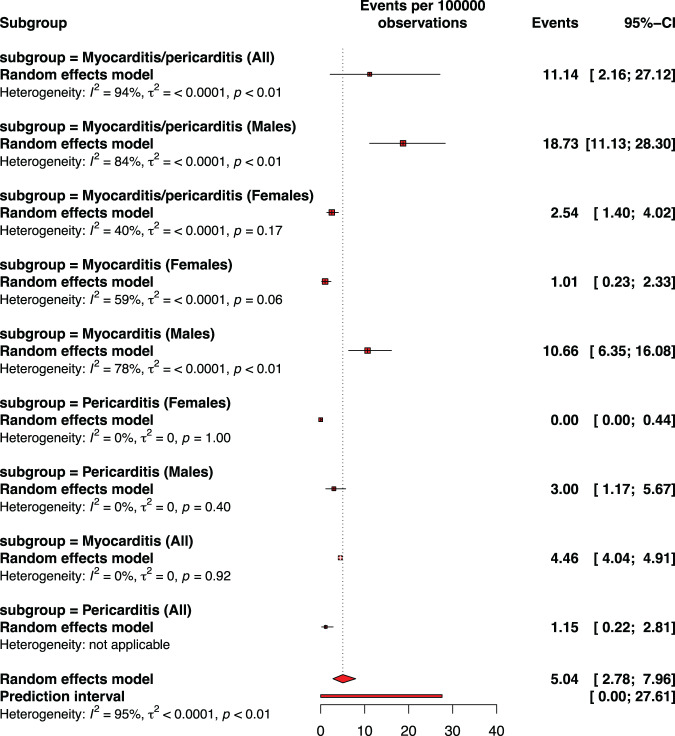
Pooled proportion of myocarditis or pericarditis or of myocarditis/pericarditis following any dose of BNT162b2 vaccination against COVID-19 among adolescents. Subgroup are presented to consider sex. The pooled incidence of studied inflammatory heart conditions following any dose was 5.04 cases per 100,000 doses of BNT162b2 vaccine. The whisker represents the 95% confidence interval. The inclusion period of participants spanned from December 2020 to February 2022.

**Fig. 5:**
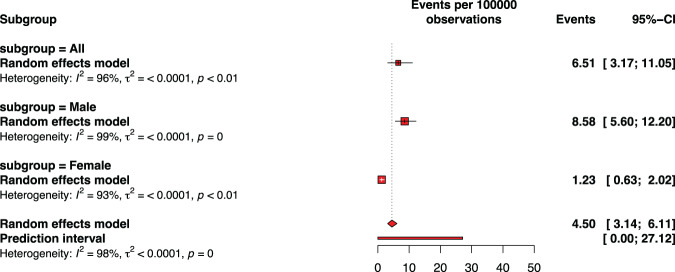
Pooled proportion of myocarditis or pericarditis or of myocarditis/pericarditis across all doses including booster dose of BNT162b2 vaccination against COVID-19 among adolescents. Subgroup are presented to consider sex. The pooled incidence of studied inflammatory heart conditions across all doses was 4.50 cases per 100,000 doses of BNT162b2 vaccine. The whisker represents the 95% confidence interval. The inclusion period of participants spanned from December 2020 to February 2022.

**Fig. 6:**
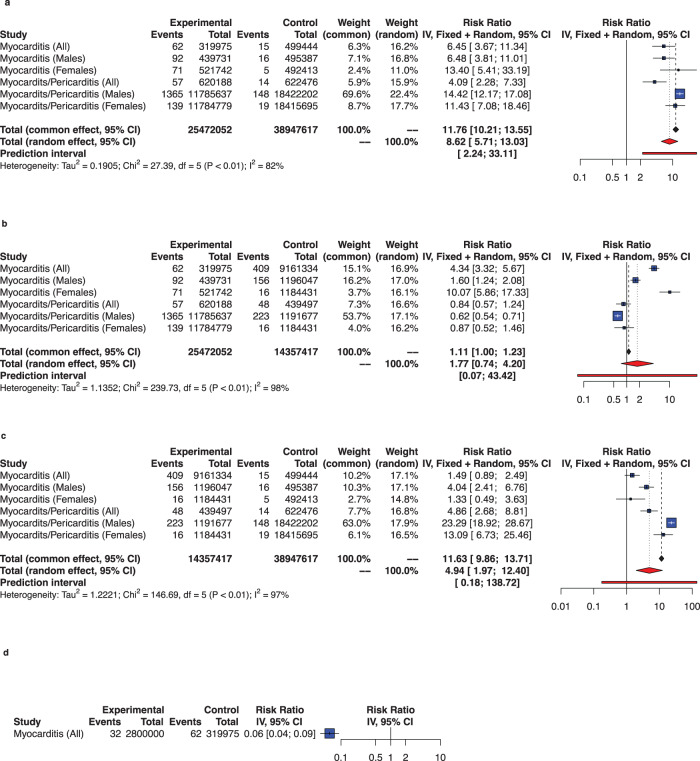
Comparing rates of myocarditis or of myocarditis/pericarditis by dose of BNT162b2 vaccination against COVID-19 among adolescents. In panel **a**, Experimental refers to second dose and control to first dose; in panel **b**, experimental refers to second dose and control to any dose, in panel **c**, experimental refers to any dose and control to first dose; in panel **d** experimental refers to booster dose and control to second dose. Conditions are presented to consider sex. The whisker represents the 95% confidence interval. The inclusion period of participants spanned from December 2020 to February 2022.

Figures 2–6 and Supplementary Fig. 2–8 describe the pooled proportion of inflammatory heart disease (myocarditis, or pericarditis, or myocarditis and pericarditis) after dose 1, 2, any dose, primary doses, and across all doses (including booster dose) of BNT162b2 among adolescents. Overall, the highest pooled incidence was observed after dose 2 (9.26 per 100,000 vaccinations [95% CI: 5.26–13.96]). The pooled incidence of inflammatory heart disease across all doses was 4.50 (95% CI: 3.14–6.11) and when only doses 1 and 2 were included was 4.63 (95% CI: 2.69–12.68) per 100,000 vaccinations. The risk of developing inflammatory heart disease was statistically significant after dose 2 versus dose 1 (RR: 8.62, 95% CI: 5.71–13.03) (Fig. 6, panels a-c). Adolescents were less likely to report cardiac inflammatory conditions after receiving a booster dose than after receiving dose 2 (RR: 0.06, 95% CI: 0.04–0.09) (Fig. 6, panel d).

### Myocarditis or pericarditis by sex

**Fig. 7:**
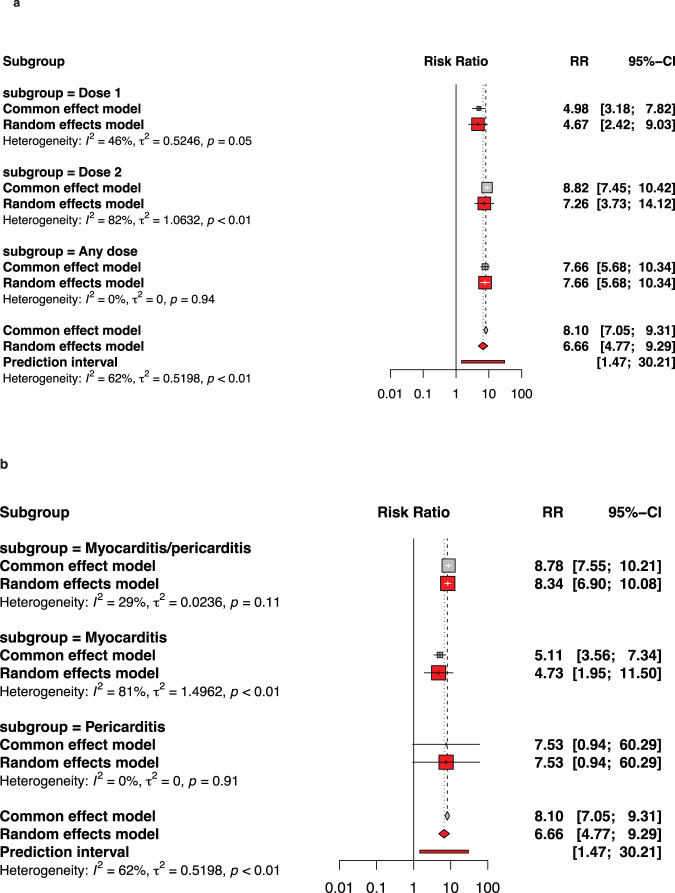
Comparing rates of myocarditis or of myocarditis/pericarditis by gender after BNT162b2 vaccination against COVID-19 among adolescents. In panel a, subgroups are by type of dose while in panel b, subgroups consider type of inflammatory heart conditions. In both panels **a** and **b**, experimental refers to males and control to females. The whisker represents the 95% confidence interval. The inclusion period of participants spanned from December 2020 to February 2022.

Figure 7 and Supplementary Fig. 9-10 describe the pooled proportion of inflammatory heart disease (myocarditis, or pericarditis, or myocarditis and pericarditis) by sex after BNT162b2 among adolescents. Across all types of doses, stratification by sex and condition (myocarditis/pericarditis or pericarditis, we found that males had the highest incidence of myocarditis (9.33 per 100,000 vaccinations [95% CI: 5.09–14.84]), whereas females had the lowest incidence of isolated pericarditis (close to 0 per 100,000 vaccinations). As a result, males were nearly seven times (RR: 6.66, 95% CI: 4.77–9.29) more likely than females to develop cardiac inflammation following BNT162b2 immunization. Similarly, males were more likely to report inflammatory heart regardless of dose, especially after any dose (RR: 7.66, 95 percent CI: 5.68–10.34) and dose 2 (RR: 7.26, 95 percent CI: 3.73–14.12). A similar tendency was detected among males in a sub-analysis by age group.

### Myocarditis or pericarditis by period length after vaccination

The pooled cumulative incidence stratified by week and dose (Supplementary Fig. 11–13) indicated that the incidence of inflammatory heart disease in adolescents following BNT162b2 vaccination varied depending on the duration used for data collection or reporting, i.e. week after dose 1 or dose 2, or booster dose. For example, studies reporting data collected within a two-week follow-up period after vaccination tended to report more inflammatory heart events than others, followed by those reporting data collected within a three-week period, then those reporting data collected within a one-week period, and finally those reporting data collected within more than three weeks period.

### Narrative summary of symptom, prognostic, reporting system and risk-benefit

In the cases where we could not locate or deduce denominators, we report our findings narratively. These studies all shared four key findings. First, myocarditis is the most often reported serious adverse event following BNT162b2 vaccination in predominantly male adolescents^13,16,19,21,22,26^, although its frequency is lower than that caused by SARS-CoV-2^13^. Second, its incidence may vary depending on the source. For example, sensitivity analysis among 12–17-year old by sex within the WHO global safety database^28^ indicated 21/296 cases of myocarditis among females when restricted to serious reports compared to 0/113 cases when restricted to healthcare professionals (Supplementary Fig. 14). Third, clinical characteristics and early results are consistent across different pediatric age groups; chest discomfort is the most reported symptom; myocardial damage and oedema is detected at cardiac magnetic resonance imaging (CMRI), and the clinical course is moderate with rapid recovery and excellent short-term outcomes. Death is uncommon, but long-term outcomes remain to be understood^16,17,21^. Fourth, the benefits of the vaccination outweigh the hazards for all age and gender groupings^13,18,19,22^.

## Discussion

### Main findings

This study examines the incidence and risk of inflammatory heart conditions, namely myocarditis, and pericarditis in adolescents following BNT162b2 vaccination against COVID-19 infection. The pooled incidence of inflammatory heart conditions was 4.5 (95% CI: 3.14–6.11) per 100,000 vaccinations across all doses, with the risk of presenting inflammatory heart disease nearly nine times higher after dose two than dosage 1. On the other hand, adolescents were found less likely to report inflammatory heart problems after receiving a booster dosage than after receiving dose 2. Male adolescents were more likely to report myocarditis across all doses (9.33 per 100,000 vaccinations [95% CI: 5.09–14.84]). They were nearly seven times more likely than female adolescents to develop any inflammatory heart condition following BNT162b2 immunization.

### Our findings in context of global rise of myocarditis

Myocarditis in children and adolescents has been linked to infectious etiologies for over seventy years^28^. The global burden of myocarditis is increasing^29^, as the number of viruses related to inflammatory heart disease has risen over time. Our findings complement previous data from large studies in the United States^6^, Israel^4^, Canada^30^, Hong Kong^24^, England^31^, as well as in multiple systematic reviews and meta-analyses on the general population^32,33^. This serious side effect is not limited to the COVID-19 vaccine or to mRNA-based vaccines. A recent review indicated that, when compared to the COVID-19 vaccine, the incidence of myopericarditis was considerably greater after smallpox vaccines and not significantly different after influenza vaccinations^32^. Patone et al.^31^. estimated an additional one, two, and six myocarditis cases per million vaccinated with BNT162b2, ChAdOx1, and mRNA-1273 in England 28 days after the first dose. They also found an additional 40 (95% CI 38–41) myocarditis per 1 million patients in the 28 days following a SARS-CoV-2 positive test. This is associated with the identification of a larger repertory of viruses throughout time, as well as the appearance of novel viruses or viral genotypes in the heart^34^. Hence, SARS-CoV-2 postinfective acute myocarditis in otherwise healthy children raises the possibility of another significant consequence of COVID-19 infection^35^, to be completely comprehended.

### Consideration for risk-benefit weighing

Important factors are that, first, all school-aged children can be affected by SARS-CoV-2 cardiovascular complications. This can be mediated by multisystem inflammatory syndrome in children (MIS-C), resulting in cardiac problems, which account for the majority of pediatric COVID-19 mortality^36^. Second, SARS-CoV-2 cardiovascular complications can negatively affect children and adolescents in settings with variable resources^37,38^. Third, the prognosis appears to be excellent in individuals with myocarditis caused by the COVID-19 vaccination. According to a proportion meta-analysis^39^, complete resolution of symptoms is achieved in 80.5 percent of patients, while the proportion of participants requiring intensive care unit admission is seven percent. In another review, patients receiving the BNT162b2 vaccination had fewer systemic symptoms and left ventricular dysfunction than mRNA1273 recipients. Those under the age of 20 exhibited higher fevers and myalgia but had a higher ejection fraction and less apparent myocardial inflammation on MRI than older patients^40^. Fourth, to rule out overreporting due to the public interest in COVID-19 immunization, the reporting method and instruments utilized to confirm myocarditis are critical. For example, following the European Medicines Agency investigation, only 14/113 patients sent to a cardiology practice for suspected myopericarditis following COVID-19 vaccination had myopericarditis-related CMRI^41^. At the global level, the WHO vigiBase^13^ reported rates of myocarditis vary significantly depending on who reports the illness. Subsequently, data sources and tools used to validate myocarditis should stay at the forefront of national and global safety surveillance, and policy should be data driven.

### Adapting policy in response to emerging evidence

So far, global strategic and adaptive policies have not been uniformed. Norway, the United Kingdom, Taiwan, and Hong Kong have halted the second dose of mRNA vaccination for adolescents. In Hong Kong^24^, for example, the single-dose regimen was linked to a lower incidence of myocarditis among vaccinated adolescents. Limitations include sample size during the post-policy era, since no local transmission of SARS-CoV-2 has occurred since May 2021, despite intensive nonpharmaceutical treatments. Canada kept both primary doses while modifying the interdose interval. As a result, in a population-based cohort study^30^ of Ontario adolescents and adults, the overall rate of myocarditis/pericarditis after BNT162b2 was significantly lower when the interdose interval was 56 or above vs 30 or fewer days. The European Union Policy^42^ puts emphasis on providing booster dose to adolescents with comorbidities. The USA has not only maintained primary vaccination that has been adapted over time to the interval dose policy, but has also supported the booster doses^20^. We showed that as compared to the second dose, the booster dose was associated with lower cardiac inflammatory events. While the interval time between doses 1 and 2 in most included studies was around 2 to 3 weeks, it is considered that the interval time for booster dose was much longer. Recent research in systems biology has indicated that interferon-gamma (INF-gamma) plays a key role in the biological processes that lead to adverse cardiac events by affecting the MAPK and JAK-STAT signalling pathways^9,10^. As a result, it is probable that the longer interval between primary vaccine doses and booster doses has contributed to a reduction in the risk of developing inflammatory adverse reactions. Finally, from modelling studies, the strategic programmatic attitude might consider the community epidemiological status^19^, clinical severity^18^ child medical history^23^, and age-gender^3^.

### Strengths, limitations, and way to improve surveillance

While our findings are unique and instructive, they should be interpreted cautiously due to several limitations. To begin, the significant heterogeneity detected between studies might be attributed to data source variability and differences in the case definition. The WHO, the CDC, and the Royal College of Pediatrics and Child Health have now supplied three case definitions for MIS-C. CMRI may be cost-effective in symptomatic adolescent boys at risk of myopericarditis following mRNA immunization. In clinical practice, endomyocardial biopsy (EMB) may be limited to suspected acute myocarditis with cardiogenic shock^34^. In settings with variable resources, additional myocarditis reporting algorithms are needed. Second, despite the temporal link with COVID-19 vaccination, the etiology of myocarditis in these individuals cannot be confirmed in most reports. Third, most studies lacked a history of SARS-CoV-2 and MIS-C, even though the risk of post-infectious myocarditis is crucial in advising practices. Fourth, data on myocarditis associated with a booster dose was only available from one passive surveillance study. A passive surveillance system is susceptible to reporting biases^1,13,41^. Firth, we did not consider socioeconomic or racial status. In terms of health equity, racial and ethnic minority groups have greater rates of COVID-19 and severe illness; prospective changes in vaccine policy, or any strategy that influences vaccination coverage for adolescents or young adults, may disproportionately harm those groups^3^. Sixth, we did not compare our findings to myocarditis baseline data. However, present evidence for background myocarditis is conflicting and limited by the use of aggregated data^43^.

Sixth, our research identifies a methodological flaw in the evidence. Several studies did not describe how vaccination status was validated, resulting in uncertainties around intervention classification and the likelihood of deviations from intended interventions. Attrition bias was difficult to determine in these observational studies due to the passive nature of data collection in observational designs, as was misclassification bias due to a lack of clear outcome definitions or adjudication. These issues usually cause unclear domain assessments. No study could compare selective outcome reporting against a prospective study registry or a priori statistical analysis plan. We acknowledge the extraordinary, often emergency conditions under which many of these investigations were done; however, future studies may benefit from careful planning and design as COVID-19 becomes endemic. Studies would be strengthened by measuring and adjusting for confounding factors. Moreover, active data collection may reduce attrition bias, albeit at a cost.

Finally, notwithstanding the low incidence of myocarditis and pericarditis following BNT162b2 vaccination, both conditions were reported, particularly in male adolescents. Policy adjustments based on new findings, such as increasing the interdose interval policy, may be recommended. To minimize overreporting, which might undermine the effectiveness of the COVID-19 vaccine and worsen vaccination inequities over the life-course, the reporting mechanism and confirmation methods must be strengthened.

## Methods

### Search strategy

This meta-analysis and systematic review complies with the PRISMA reporting guidance. From 2020 through May 2022, three online bibliographic databases were searched: EMBASE, MEDLINE via PubMed, and Cochrane CENTRAL. A search technique was built in PubMed and iteratively modified to enhance sensitivity and specificity for identifying relevant publications. Following this stage, key search terms were developed and comprised three core groups of terms relevant to inflammatory heart conditions: myocarditis, myopericarditis, and pericarditis. Using Boolean operators, a range of terms associated with each of these MeSH terms related to “BNT162b2 vaccination” AND “adolescents” were entered into each database. No geographical or language restrictions were applied. We manually evaluated the references of included articles and associated systematic reviews to identify any other relevant research. We did not include preprint publications. Instead, relevant studies were located by searching the New England Journal of Medicine, Nature, and The Lancet COVID-19 databases.

### Screening, eligibility, and data management

After the cleanup of duplicate records (PK), two reviewers (LB and JLT) independently assessed the relevance of the titles and abstracts before uploading and selecting relevant full papers in Covidence (Veritas Health Innovation, Melbourne, Australia. Available at www.covidence.org.). A third author (PK) confirmed the validity of the included studies. Disagreements were handled a debate through consensus at each level until consensus was reached; alternatively, a third author (PK) offered arbitration. This analysis excluded case reports, case series with fewer than 25 individuals, and reviews. Furthermore, we excluded studies that did not report findings on the age group of interest, as well as those that assessed overall study results on mRNA vaccines without defining the type of mRNA vaccination. However, we opted to include for narrative synthesis two papers in which estimates were not provided by the type of mRNA vaccination, but more than half of the study participants were adolescents who received the BNT162b2 vaccine. We included studies with an upper age limit of no more than 19 years for large studies, such as those undertaken in Israel, where the cutoff was higher than our threshold age range of 12 to 17 years. Three authors (MK, JLT, and LB) extracted data using a specialized form to record the study identification (year of publication, period of data collection, and country), study design, study sample, and type of reported measures of association (age group, sex, relative risk, risk ratio, and incidence per number of observations/ vaccinations), study outcome (type of inflammatory heart condition and associated severity), and study conclusion. Each study included cases of myocarditis, myopericarditis, and pericarditis were documented individually and described here as inflammatory heart disease. To address missing or unclear estimates of interest (such as the denominator used to calculate the incidence or the incidence among adolescents aged 12 to 17 years), we used an online calculator (https://www.omnicalculator.com/health/incidence-rate) to convert available estimates. Otherwise, we contacted the corresponding author of the included study via email for clarification. Four papers were excluded because the authors could not be reached or did not offer an adequate response.

### Assessment of risk of bias in included studies

Two reviewers (LB and MK) assessed the risk of bias (RoB) of each included study using the ROBINS-I tool^44^ for non-randomized studies. One senior reviewer with Cochrane experience (AB) conducted all RoB assessments as the independent duplicate reviewer. Standard domains for each tool were used, and an overall RoB was determined using a worst-domain scenario approach (Supplementary Table 2 and Supplementary Table 3). As a result, we judged the overall RoB for each study as ‘low risk’, ‘unclear risk’ or ‘high risk’ and reported the main reason in the summary table for RoB. Any discrepancies in individual domain judgments as well as overall judgments were resolved through consensus or adjudication by a third review author (PK).

### Data analysis

We analyzed quantitative data using the *meta, metaprop* and *metabin* packages (RevMan5 layout) of the statistical software *R* (version 4.0.3, R Foundation for Statistical Computing, Vienna, Austria). We performed a random-effects meta-analysis of the incidence of myocarditis and pericarditis following BNT162b2 in adolescents using the inverse variance approach and reported the related prediction intervals. We conducted sub-analyses by dose (first, second, booster, or any dose), sex (male, female, and all), and age group (12–15, 16–17, 16–19 years, and all ages) and calculated the cumulative pooled incidence by week (seven-day interval). Pooled dichotomous data were expressed as risk ratios (RR) with 95% confidence intervals (CIs) for comparisons of myocarditis and pericarditis in adolescents after the primary dose, i.e., the dose 2 vs. dose 1, or any dose (here considered as control), and the booster dose vs. primary doses (here considered as control), as well as between males and females (here considered as control) across different doses of BNT162b2. We used the *metabin* function to yield both fixed and random effects. However, we considered interpreting the latter due to the predicted heterogeneity between studies. Heterogeneity was assessed using the *χ*^2^ test on Cochrane’s Q statistic and quantified by I^2^ values. The I^2^ statistic measures the proportion of overall variance attributable to genuine differences across studies as opposed to random variation. We investigated small-study impact using funnel plots and tests of funnel plot asymmetry (Egger’s linear regression test), with bias correction using trim-and-fill methods. All statistical tests were two-tailed, with a *p*-value of 0.05 indicating statistical significance.

We provided a narrative summary of the data from studies that published estimates without clearly identifying the vaccination recipients or the administered dose. Moreover, risk-benefit modelling results were also presented in narrative form.

### Data sharing

All data generated or analyzed during this study are included in this published Article and the appendix.

### Reporting summary

Further information on research design is available in the Nature Research Reporting Summary linked to this article.

## Supplementary information

Supplement

REPORTING SUMMARY
