# Supplementary material for: Systematic review and meta-analysis of myocarditis and pericarditis in adolescents following COVID-19 BNT162b2 vaccination
Source: NPJ Vaccines. 2023 Jun 9;8:89. doi: 10.1038/s41541-023-00681-3 (PMC10252172; doi:10.1038/s41541-023-00681-3)
Supplement: Supplementary file 1 — Supplement [file 41541_2023_681_MOESM1_ESM.pdf]

Myocarditis and Pericarditis in Adolescents Following COVID-19 BNT162b2 Vaccination: A Systematic Review and Meta-Analysis

Supplementary Materials

Table of contents

Supplementary Table 1: Characteristic of Studies Assessing the Risk of Myocarditis/ pericarditis after COVID-19 Pfizer-BioNTech (BNT162b2) mRNA Vaccination in Adolescents.

Supplementary Table 2: Risk of bias of included studies assessed with ROBINS-I (domains 1-5)

Supplementary Table 3: Risk of bias of included studies assessed with ROBINS-I (domains 6-9)

Supplementary Figure 1: Map showing geographical origins of included studies.

Supplementary Figure 2: Pooled proportion of myocarditis or of myocarditis/pericarditis following the first dose of BNT162b2 vaccination against COVID-19 among adolescents.

Supplementary Figure 3: Pooled proportion of myocarditis or of myocarditis/pericarditis following the second dose of BNT162b2 vaccination against COVID-19 among adolescents.

Supplementary Figure 4: Pooled proportion of myocarditis or pericarditis or of myocarditis/pericarditis following any dose of BNT162b2 vaccination against COVID-19 among adolescents.

Supplementary Figure 5: Pooled proportion of myocarditis or pericarditis or of myocarditis/pericarditis across all doses including booster dose of BNT162b2 vaccination against COVID-19 among adolescents.

Supplementary Figure 6: Pooled proportion of myocarditis or pericarditis or of myocarditis/pericarditis stratified by dose of BNT162b2 vaccination against COVID-19 among adolescents.

Supplementary Figure 7: Pooled proportion of myocarditis or of myocarditis/pericarditis following primary doses of BNT162b2 vaccination against COVID-19 among adolescents.

Supplementary Figure 8: Comparing rates of myocarditis or of myocarditis/pericarditis by gender after BNT162b2 vaccination against COVID-19 among adolescents.

Supplementary Figure 9: Pooled proportion of myocarditis or pericarditis or of myocarditis/pericarditis stratified by sex after BNT162b2 vaccination against COVID-19 among adolescents.

Supplementary Figure 10: Comparing rates of myocarditis or of myocarditis/pericarditis by gender after BNT162b2 vaccination against COVID-19 among adolescents.

Supplementary Figure 11: Pooled cumulative proportion of myocarditis or pericarditis or of myocarditis/pericarditis before and after seven reporting days of BNT162b2 vaccination against COVID-19 among adolescents.

Supplementary Figure 12: Pooled cumulative proportion of myocarditis or pericarditis or of myocarditis/pericarditis after each seven days period across all doses including booster dose of BNT162b2 vaccination against COVID-19 among adolescents.

Supplementary Figure 13: Pooled cumulative proportion of myocarditis or pericarditis or of myocarditis/pericarditis after each seven days period after the first (Panel A) and the second dose (Panel B) of BNT162b2 vaccination against COVID-19 among adolescents.

Supplementary Figure 14: Reporting of myopericarditis among adolescents aged 12-17 years using data from the passive surveillance, nested in the World Health Organisation database (VigiBase) of spontaneous reports.

Supplementary Table 1: Characteristic of Studies Assessing the Risk of Myocarditis/ pericarditis after COVID-19 Pfizer-BioNTech (BNT162b2) mRNA Vaccination in Adolescents.

Author, Year, Design and Timeline	Age Group	Time between vaccination and myocarditis event	Dose Type	Sex	Condition	Estimates	Estimates in vaccinated	Low Limit	Upper Limit	Author conclusion
**Block et al. 2022[1], US, Cohort study, hospital based, January 1- 2021-January 31, 2022; 13 months**	12–17 years	7 days	1	M	MD	RR	23	5.3	99.5	*Data from 40 health care systems participating in a large network<br>*Risk for cardiac complications<br>significantly higher after SARS-CoV-2 infection than after mRNA COVID-19 vaccination for both males and females in all age groups.
	12–17 years	21 days	1	M	MD	RR	18	5.4	60.6	
	12–17 years	7 days	2	M	MD	RR	2.3	1.2	4.4	
	12–17 years	21 days	2	M	MD	RR	2.2	1.2	4	
	12–17 years	7 days	Any	M	MD	RR	3.9	2.1	7	
	12–17 years	21 days	Any	M	MD	RR	3.7	2.1	6.4	
	12–17 years	7 days	1	M	M/P	RR	25.7	6	110.3	
	12–17 years	21 days	1	M	M/P	RR	19.8	5.9	66.2	
	12–17 years	7 days	2	M	M/P	RR	2.1	1.1	3.9	
	12–17 years	21 days	2	M	M/P	RR	1.8	1	3.1	
	12–17 years	7 days	Any	M	M/P	RR	3.5	2	6.1	
	12–17 years	21 days	Any	M	M/P	RR	3	1.8	5	
	12–17 years	7 days	1	F	MD	RR	24.5	3.1	193.3	
	12–17 years	21 days	1	F	MD	RR	35.4	4.6	270.5	
	12–17 years	7 days	2	F	MD	RR	23.1	2.9	182	
	12–17 years	21 days	2	F	MD	RR	11.1	3.2	39	
	12–17 years	7 days	Any	F	MD	RR	31.2	6.7	144.3	
	12–17 years	21 days	Any	F	MD	RR	18	6.4	50.5	
	12–17 years	7 days	1	F	M/P	RR	12.2	2.6	56.7	
	12-17 years	21 days	1	F	M/P	RR	17.7	4	78.4	
	12–17 years	7 days	2	F	M/P	RR	11.5	2.5	18.7	
	12–17 years	21 days	2	F	M/P	RR	6.7	2.4	18.7	
	12–17 years	7 days	Any	F	M/P	RR	15.6	4.8	50.6	

	12–17 years	21 days	Any	F	M/P	RR	10	4.3	23.4	
**Chua et al., 2022[2], China, Prospective cohort, 14 June 2021 and 4 September 2021**	12-17 years				MD	RR				*Significant increase in the risk of acute myocarditis/pericarditis following Comirnaty vaccination among *Chinese male adolescents, especially after the second dose.
	12-17 years	14 days	1	M	M/P	RD	5.36	0.65	10.07	
	12-17 years	14 days	1	F	M/P	RD				
	12-17 years	14 days	2	M	M/P	RD	37.1	25.1	49.12	
	12-17 years	14 days	2	F	M/P	RD				
	12-17 years	14 days	1	All	M/P	RD	3.26	-0.4	6.92	
	12-17 years	14 days	2	All	M/P	RD	21.1	12.06	30.16	
	12-17 years	14 days	Any	All	M/P	RD	18.4	9.95	26.87	
	12-17 years	14 days	Any	M	M/P	RD	32.1	20.91	43.25	
	12-17 years	14 days	Any	F	M/P	RD				
	12-17 years	28 days	1	M	M/P	RD	6.72	1.13	12.31	
	12-17 years	28 days	1	F	M/P	RD				
	12-17 years	28 days	2	M	M/P	RD	38.2	25.85	50.51	
	12-17 years	28 days	2	F	M/P	RD				
	12-17 years	28 days	1	All	M/P	RD	4.14	-0.17	8.45	
	12-17 years	28 days	2	All	M/P	RD	22.4	13.01	31.87	
	12-17 years	28 days	Any	All	M/P	RD	19.3	10.53	28.05	
	12-17 years	28 days	Any	M	M/P	RD	32.7	21.26	44.16	
	12-17 years	28 days	Any	F	M/P	RD				
**Chouchana et al., 2022[3], WHO countries (VigiBase), Passive surveillance, nested in a database of spontaneous reports**	12-17 years	any	Any	M	MD	ROR	18.5	15.6	21.9	*Inflammatory heart reactions may rarely occur shortly following mRNA COVID-19 vaccination. * An important disproportionate reporting of myocarditis among adolescents' male patients, *But reporting rates support a) a very rare risk, that does not seem to compromise the largely positive benefit-risk balance of these vaccines and b) a very rare incidence, lower than reports of myocarditis related to severe COVID-19 infection
	12–17 years	any	Any	F	MD	ROR	7.8	5.1	12	
	12–17 years	any	Any	M	PD	ROR	3.7	2.6	5.1	
	12–17 years	any	Any	F	PD	ROR	0.8	0.3	2.3	
	12–17 years	any	Any	All	MD	ROR	22.3	19.2	25.9	
	12–17 years	any	Any	All	PD	ROR	3.7	3.2	4.2	

**registered up to 30 June 2021**										
**Karlstad et al., 2022[4], Denmark, Finland, Norway, and Sweden, cohort study, December 27, 2020 to October 5, 2021**	12 years or older.	28 days	Any	M	MD	IRR	1.4	1.09	1.8	*A large cohort study; both first and second doses of mRNA vaccines associated with increased risk of myocarditis and pericarditis.<br>*Findings compatible with between 4 and 7 excess events in 28 days per 100 000 vaccinees after BNT162b2<br>*Risk to be balanced against the benefits of protecting against severe COVID-19 disease.
	12 years or older	28 days	Any		MD	IRR	1.46	1.01	2.11	
**Li et al., 2022[5], Hong Kong-China, Prospective cohort study, population based, March 10 and October 18, 2021**	12-17 years		1	All	MD	Risk per 100.000	3.12			*Single-dose regimen associated with reduction in myocarditis risk among vaccinated adolescents.
	12-17 years		2	All	MD	Risk per 100.000	22.2			
	12-17 years		1	M	MD	Risk per 100.000	5.27			
	12-17 years		2	M	MD	Risk per 100.000	39			
	12-17 years		1	F	MD	Risk per 100.000	0.9			
	12-17 years		2	F	MD	Risk per 100.000	4.97			
**Funk et al., 2022[6], United states of America, simulation study with sensitivity analysis of the benefits and risks of the vaccine across possible**	16–17 years	any	Any	M	M/P	Risk per 100.000	42.9			*Benefits of the vaccine outweigh its risks for all age and sex subgroups we analyze in this study.<br>*Uncertainties exist in this assessment as both benefits and risks of vaccination may change with the continuing evolution of the pandemic.
	16–17 years	any	Any	F	M/P	Risk per 100.000	47.9			
	16–17 years	any	Any	M	M/P	Rate of excess	196	36	424	

**pandemic scenarios**						mortality				
	16–17 years	any	Any	F	M/P	Rate of excess mortality	36	0	298	
	16-17 years	any		M	M/P	Rate of excess mortality	196	36	424	
	16-17 years			F	M/P		36	0	298	
**Krug et al., 2022[7], USA, passive surveillance, 1 January 2021 to 18 June 2021**	12-15 years	7 days	1	M	M/P	Risk per 100.000	1.14			*Findings strongly support individualized pediatric COVID-19 vaccination strategies which weigh protection against severe disease vs. risks of vaccine-associated myo/pericarditis. *Research needed into the nature and implications of this adverse effect and immunization strategies which reduce harms in this overall low risk cohort.
	12-15 years	7 days	2	M	M/P	Risk per 100.000	16.2			
	12-15 years	7 days	1	F	M/P	Risk per 100.000	0			
	12-15 years	7 days	2	F	M/P	Risk per 100.000	1.3			
	16-17 years	7 days	1	M	M/P	Risk per 100.000	0.82			
	16-17 years	7 days	2	M	M/P	Risk per 100.000	9.3			
	16-17 years	7 days	1	F	M/P	Risk per 100.000	0.14			
	16-17 years	7 days	2	F	M/P	Risk per	1.25			

						100.000		
	12-17 years	7 days	1	M	M/P	Risk per 100.000	0.1	
	12-17 years	7 days	2	M	M/P	Risk per 100.000	11.9	
	12-17 years	7 days	1	F	M/P	Risk per 100.000	0.06	
	12-17 years	7 days	2	F	M/P	Risk per 100.000	1.27	
**Nygaard et al., 2022[8], Denmark, Prospective population-based cohort study, May 15 to September 15, 2021**	12–17 years		1	M	M/P	Risk per 100.000	9.7	*Incidence of myopericarditis after COVID-19 vaccination among males appears higher than reports from the United States.
	12–17 years		1	F	M/P	Risk per 100.000	1.6	
**Oster et al., 2022[9], United State of America, passive surveillance, December 2020 to August 2021**	12-15 years		1	M	MD	Risk per 100.000	0.71	*Based on passive surveillance reporting in the US, the risk of myocarditis after receiving mRNA-based COVID-19 vaccines increased across multiple age and sex strata and highest after the second vaccination dose in adolescent males and young men.<br>*Risk to be considered in the context of the benefits of COVID-19 vaccination
	12-15 years		2	M	MD	Risk per 100.000	7.1	
	12-15 years		1	F	MD	Risk per 100.000	0.05	
	12-15 years		2	F	MD	Risk per 100.000	0.64	
	16-17 years		1	M	MD	Risk per	0.72	

					100.0 00		
	16-17 years	2	M	MD	Risk per 100.0 00	10.6	
	16-17 years	1	F	MD	Risk per 100.0 00	0.08	
	16-17 years	2	F	MD	Risk per 100.0 00	1.1	
**Yap et al., 2022[10], Singapore, Retrospective review of HSA vaccine adverse event (Passive surveillance), 21 June 2021- 25 July 2021**	12-19 years	Any	M	MD	Risk per 100.0 00	3.72	*Data from the national registry in Singapore indicate an increased incidence of pericarditis and myocarditis in younger men after COVID-19 mRNA vaccination
	12-19 years	Any	F	MD	Risk per 100.0 00	0	
	12-19 years	Any	All	MD	Risk per 100.0 00	1.91	
	12-19 years	Any	M	PD	Risk per 100.0 00	1.11	
	12-19 years	Any	F	PD	Risk per 100.0 00	0	
	12-19 years	Any	All	PD	Risk per 100.0 00	0.57	
	12-19 years	Any	M	M/P	Risk per 100.0 00	4.8	
	12-19 years	Any	F	M/P	Risk per	0	

						100.000				
	12-19 years		Any	All	M/P	Risk per 100.000	2.48			
**Das et al., 2021[11], United States of America, Retrospective cohort, May 10, 2021, and June 20, 2021**	12-18 years	any	Any		M/P	Risk per 100.000				*Data suggest that symptoms owing to myopericarditis after the mRNA COVID-19 vaccination tend to be mild and transient.
**Foltran et al., 2021[12], VigiBase/WHO, Retrospective review (Passive surveillance), 1 January 2021 and 14 September 2021**	12-17 years	any	Any	All	M/P	Risk per 100.000	5			*This investigation including only adolescent data suggests for the first time that the second dose of mRNA COVID-19 vaccines increases the risk of reporting myocarditis/pericarditis compared with the first dose particularly in boys without significant difference between tozinameran and elasomeran.
	12-17 years	any	2vs1	All	MD	ROR	4.98	3.05	8.27	
	12–17 years	any	2vs1	All	PD	ROR	5.44	2.01	16.1	
	12–17 years	any	2vs1	All	M/P	ROR	4.95	3.14	7.89	
	12–17 years	any	Any	Mvs F	MD	ROR	14.9	7.63	33.6	
	12–17 years	any	1	Mvs F	M/P	ROR	10.1	4.26	29.6	
	12–17 years	any	2	Mvs F	M/P	ROR	10.2	4.88	25	
**Gurdasani et al., 2021[13], United Kingdom, Risk-benefit analysis/modelling, 1 July 2020 and 31 March 2021.**	12–17 years		1	M	M/P	Risk per 100.000	0.7			*Given the current (as of 15 September 2021) high case rates (680/100,000 population/week in 10–19-year-old) in England, findings support vaccination of adolescents against SARS-CoV2.
	12–17 years		2	M	M/P	Risk per 100.000	6.3			
	12–17 years		1	F	M/P	Risk per 100.000	0			
	12–17 years		2	F	M/P	Risk per 100.000	0.9			

	12–17 years		Any	All	M/P	Death	4			
	12–17 years				M/P	Death	0			
**Dagan, M.D., 2021[14], Tel Aviv, Israel, target trial emulation, December 20, 2020, to February 1, 2021**	16-39 years	42 days	Any		MD	RR				*Risk of myocarditis, increased after both vaccination and SARS-CoV-2 infection. *After vaccination, the risk increased mostly among young male adolescents and adults (16 to 39 years of age), with 8.62 excess events per 100,000 persons *After infection, the risk was increased in both age categories (<40 and ≥40 years) and in both male and female adolescents and adults, with 11.54 excess events per 100,000 persons in young male adolescents and adults
	16-39 years		Any	M	MD	RR	8.62	2.8	14.35	
	16-39 years		Any	F	MD	RR	-0.63	-1.9	0	
	16-39 years		Any		PD	RR				
	16-39 years		Any	M	PD	RR	5.28	0.17	10.33	
	16-39 years		Any	F	PD	RR	0.54	0	1.89	
**Hause 2021[15], USA, passive and active surveillance data, December 14, 2020– July 16, 2021**	12-17 years		Any	All	M/P	Reported proportion	40% of serious events reported			Local and systemic reactions after vaccination with Pfizer-BioNTech vaccine were commonly reported by adolescents aged 12–17 years to U.S. vaccine safety monitoring systems, especially after dose 2. A small proportion of these reactions are consistent with myocarditis
**Hause 2022[16], USA, passive and active surveillance, December 9, 2021–February 20, 2022**	12-17 years		3	All	M/P	Reported proportion	61% of serious events reported			*Myocarditis, less frequently reported after a booster dose than a second primary dose.
**Mevorach 2022[17], Israel, retrospective cohort, December 20, 2020, to May 31, 2021**	16-19 years	21 days	2vs1	M	MD	RD	13.7	8.11	19.46	*Incidence of myocarditis, although low, increased after the receipt of the BNT162b2 vaccine, particularly after the second dose among young male recipients. *Clinical presentation of myocarditis after vaccination usually mild.
	16-19 years	21 days	2vs1	F	MD	RD	1	-0.63	2.72	
**Jain 2021[18], USA, Retrospective multicenter study across 16 US hospital, March 2021 and June 2021**	<21 years, average 15.6 years, range 12-20 years., 94% received BNT162b2	14 days	Any	All	M/P	Death	0			*Clinical characteristics and early outcomes, similar between different pediatric age groups in C-VAM. *Hospital course is mild, with quick clinical recovery and excellent short-term outcomes. *Myocardial injury and edema noted on CMR. *Close follow-up and further studies needed to understand long-term implications and mechanism of these myocardial tissue changes.

**June-Choe 2021[19], South Korea, Retrospective cohort, from July 2021-?**	From 10th grade to 12th grade (around 16–18 years of age)	21 days	1	All	M/P	Risk per 100.000	1.8			*BNT162b2 vaccination was safe and associated with a significantly lower risk of SARS-CoV-2 infection, *Vaccination in adolescent may reduce the burden of Covid-19
		30 days	2	All	M/P		4.3			
**Lai 2021[20], Hong Kong, Population-based retrospective cohort,**	Adolescents aged 12–18 with a territory-wide electronic health care database	28 days	1	All	MD	IRR	9.15	1.14	73.16	*Overall absolute risk of AESIs was low with no evidence of an increased risk of AESIs except myocarditis and sleeping disturbances/disorders.
		28 days	2	All	MD		29.61	4.04	217.07	
**Karlstad 2022[4], Denmark, Finland, Norway, and Sweden, Retrospective population-based cohort, December 2020-October 2021**	General population, here data of 12-15 years are reported (++ received BNT162b2)	21 days	1	M	MD	IRR	4.77	1.85	12.26	*Increased risk of myocarditis /pericarditis after both dose 1 and 2 of mRNA vaccine. *For individuals receiving 2 doses of the same vaccine, risk of myocarditis highest among young males (aged 16-24 years) after dose 2. *Findings compatible with between 4 and 7 excess events in 28 days per 100 000 vaccinees after BNT162b2, and between 9 and 28 excess events per 100 000 vaccinees after mRNA-1273. *Risk to be balanced against the benefits of protecting against severe COVID-19 disease.
			2	M	MD	IRR	13.86	5.78	33.22	

Abbreviation: M: male, F: female, MD: myocarditis, PD: pericarditis, M/P: myocarditis and pericarditis, RR: risk ratio, IRR: incidence rate ratio, ROR: reported odds ratio, RD: risk difference, C-VAM: coronavirus disease 2019 vaccination associated myocarditis, CMR: cardiovascular magnetic resonance

Supplementary Table 2: Risk of bias of included studies assessed with ROBINS-I (domains 1-5)

Study	Bias due to confounding		Bias in selection of participants		Bias in classification of interventions		Bias due to deviations from intended interventions		Bias due to missing data	
	Judgment	Comment	Judgment	Comment	Judgment	Comment	Judgment	Comment	Judgment	Comment
**Block 2022**	Unclear	'…data were obtained using a query that returned aggregate data from sites, precluding adjustment for potential confounders.' 'Stratification by age and sex was performed because of their clear prior association with cardiac outcomes.'	Low	'This study used EHR data from 40 health care systems participating in PCORnet, the National Patient-Centred Clinical Research Network…' to calculate incidences of cardiac outcomes. Cohort exclusions and index dates for cohort entrance appeared reasonable.	Unclear	'…only…mRNA COVID-19 vaccinations documented in EHRs were available for assessment.' 'Under-ascertainment of…mRNA COVID-19 vaccinations reduced sample size and might have introduced bias if capture of…vaccination within the EHR occurred differentially for those with cardiac outcomes.'	Low	Given the electronic health records this study is based on, it is unlikely that adolescents receiving or not receiving the vaccine would not have been classified as such.	Unclear	Due to the passive surveillance conducted in this study, it is impossible to determine the number of adolescents who received the vaccine and who had myocarditis events that were not reported. '…outcomes were rare in some cohorts, leading to wide CIs around RR estimates.'
**June Choe 2021**	High	Estimates were unadjusted because of the 'lack of demographic data for individual cases.	High	Adverse events following immunisation were recorded using passive surveillance, but it is not clear if these were linked to vaccination records in this cohort study.	Low	Passive surveillance for adverse events following immunisation was conducted through the Korea Immunization Management System (KIMS).	Low	Given the electronic vaccination management system this study is based on, it is unlikely that students receiving or not receiving the vaccine would not have been classified as such.	Unclear	Due to the passive surveillance conducted in this study, it is impossible to determine the number of students who received the vaccine and who had myocarditis events that were not reported.
**Chouchana 2022**	Unclear	'We performed the main analysis and a secondary analysis stratified according to age groups and sex.' No other adjustment for confounding is reported.	Low	Data were captured as part of the VigiBase database of the WHO, a global database of individual case safety reports and linked to the COVID-19 Data Tracker for assessment of the	Low	Vaccination status was linked to COVID-19 Data Tracker from the US Centres for Disease Control and Prevention.	Low	Considering the observational nature of the study and inclusion of only those who had received the vaccine, this bias is unlikely to have been introduced.	Unclear	Due to the passive surveillance conducted in this study, it is impossible to determine the number of adolescents who received the vaccine and who had myocarditis events that were not reported.

				number of people vaccinated in the US.					'Spontaneous reports will likely feature under-reporting of total real-world cases as well as variable data quality, all of which are inherent to any pharmacovigilance system.'
**Chua 2021**	High	No adjustment for confounding. Subgroup analyses were conducted by sex.	Low	'All individuals receiving the Comirnaty vaccine have also consented to their vaccination records being linked to their corresponding comprehensive electronic health records held by the Hospital Authority (HA), the major publicly funded healthcare provider, through the CARE program.'	Low	Passive surveillance for adverse events following immunisation was conducted through the vaccination records of participants, provided by the Department of Health in Hong Kong.	Low	Given the electronic vaccination records this study is based on, it is unlikely that adolescents receiving or not receiving the vaccine would not have been classified as such	Unclear	'…asymptomatic subjects and subjects with transient and subtle symptoms of acute myocarditis/pericarditis, such as tachycardia and mild chest discomfort, might not seek medical consultation or have sought medical consultation in the private sector which were not reported.'
**Dagan 2021**	Unclear	Results stratified by age and sex are reported. '…vaccinated persons and unvaccinated controls were exactly matched on a set of baseline variables that were deemed to be potential confounders according to domain expertise…' '…persons in the study were not randomly assigned	Low	No bias in the selection of participants was observed, with all CHS members of relevant age included. The exception was a number of exclusions on methodological grounds (distinguishing true new events and minimising confounders from special populations) which were	Low	Misclassification of interventions is unlikely, given that vaccination status of patients was recorded in the electronic medical record of the insurer.	Low	Given the electronic medical record data this study is based on, it is unlikely that participants receiving or not receiving the vaccine would not have been classified as such.	Unclear	Due to the passive surveillance conducted in this study, it is impossible to determine the number of adolescents who received the vaccine and had unreported myocarditis events.

		according to exposures…this may have introduced confounding at baseline and selection bias at censoring, especially since a single set of confounders was used for adjustment in the assessment of many disparate adverse events.’		considered reasonable.				
**Das 2021**	High	No adjustment for potential confounders is reported.	Low	‘Children were included in the study if they presented with probable myopericarditis after mRNA COVID-19 vaccination between May 10, 2021, and June 20, 2021, and were aged 12-18 years.’	Unclear	It is not reported how vaccination status was verified.	Unclear	It is not clear how vaccination status was verified, therefore bias due to deviations from intended interventions could not be ruled out. ‘Because the Pfizer-BioNTech (BNT162b2) vaccine is the only mRNA vaccine currently approved in individuals at 12-15 years of age, we have presumed that all patients received this vaccine (without verifying vaccination cards).’	Unclear	Due to the passive surveillance conducted in this study, it is impossible to determine the number of adolescents who received the vaccine and had unreported myocarditis events. ‘… although the patients who were symptomatic and sought medical care were evaluated and diagnosed, several COVID-19 vaccine-associated myocarditis episodes could be subclinical or have trivial symptoms that did not lead to seeking medical care.’
**Foltran 2022**	Low	‘The logistic regression model performed for the disproportionality analysis was adjusted for five variables: age, sex, type of reporter, completeness of	Low	Data were captured as part of the VigiBase database of the WHO, a global database of individual case safety reports with mRNA COVID-19 vaccines. ‘All	Unclear	It is not reported how vaccination status was verified.	Unclear	It is not clear how vaccination status was verified, therefore bias due to deviations from intended interventions could not be ruled out.	Unclear	Due to the passive surveillance conducted in this study, it is impossible to determine the number of adolescents who received the vaccine and who had

		individual case safety reports, and number of coreported drugs when the headcount allowed it.’		adolescents (12–17 years) who received mRNA COVID-19 vaccines were included.’					myocarditis events that were not reported.
**Funck 2022**	High	No adjustment for potential confounders is reported.	Low	‘Biologics Effectiveness and Safety (BEST) database and the Vaccine Safety Datalink (VSD) to monitor the incidence of myocarditis and pericarditis cases post-vaccination since EUA approval of the COVID-19 vaccines.’	Low	Vaccination status was captured as part of the Vaccine Safety Datalink (VSD).	Low	Considering the data modelling nature of the study and inclusion of only those who had received the vaccine, this bias is unlikely to have been introduced.	Unclear	Due to the passive surveillance data used in this study, it is impossible to determine the number of adolescents who received the vaccine and who had myocarditis events that were not reported.
**Gurdasani 2021**	High	No adjustment for potential confounders is reported.	Low	‘Patients were included in the analyses if they resided in England, were alive on the start date of the study period, registered with a primary care practice, had a valid pseudo-identifier for linkage and at least 28 days of follow-up.’	Unclear	It is not reported how vaccination status was verified.	Unclear	It is not clear how vaccination status was verified, therefore bias due to deviations from intended interventions could not be ruled out.	Unclear	Due to the passive surveillance conducted in this study, it is impossible to determine the number of adolescents who received the vaccine and who had myocarditis events that were not reported.
**Hause 2021**	High	No adjustment for potential confounders is reported.	Low	Data were captured as part of the Vaccine Adverse Event Reporting System (VAERS) for the COVID-19 vaccine.	Low	Vaccination status was linked to VAERS.	Low	Considering the observational nature of the study and recruitment of only those who had received the vaccine, this bias is unlikely to have been introduced.	Unclear	Due to the passive surveillance conducted in this study, it is impossible to determine the number of adolescents who received the vaccine and who had myocarditis events that were not reported.

**Hause 2022**	Low	The model on which the estimation of odds ratios is based adjusted for demographic variables and accounted for repeated measures among doses reported by each registrant.	Low	Data were captured as part of the Vaccine Adverse Event Reporting System (VAERS) for the COVID-19 vaccine.	Low	Vaccination status was linked to VAERS.	Low	Considering the observational nature of the study and recruitment of only those who had received the vaccine, this bias is unlikely to have been introduced.	Unclear	Due to the passive surveillance conducted in this study, it is impossible to determine the number of adolescents who received the vaccine and who had myocarditis events that were not reported.
**Jain 2021**	High	No adjustment for potential confounders is reported.	Low	'Patients ≤21 years of age with a diagnosis of acute myocarditis based on clinical presentation, abnormal biomarkers, and/or cardiovascular imaging findings within 2 weeks of COVID-19 vaccination were included.' Exclusions related to myocarditis with plausible alternative aetiology was considered reasonable.	Low	Vaccination status was linked to VAERS.	Low	Considering the observational nature of the study and recruitment of only those who had received the vaccine, this bias is unlikely to have been introduced.	Unclear	Due to the passive surveillance conducted in this study, it is impossible to determine the number of adolescents who received the vaccine and who had myocarditis events that were not reported.
**Karlstad 2022**	Low	Poisson regression adjusting for the following: sex, age, calendar period, health care worker status, nursing home resident, and 5 comorbidities (pulmonary disease, kidney disease, autoimmune disease, cardiovascular dis-	Low	'We included all persons who turned 12 years or older in 2021, were residents on January 1, 2017, and were alive and still residing within the country on December 27, 2020.' Exclusions for myocarditis cases prior to and until 26 December 2020 were	Low	Vaccination status was checked using the nationwide health registers on SARS-CoV-2 vaccination.	Low	Given the electronic vaccination records this study is based on, it is unlikely that adolescents receiving or not receiving the vaccine would not have been classified as such.	Unclear	Due to the passive surveillance conducted in this study, it is impossible to determine the number of adolescents who received the vaccine and who had myocarditis events that were not reported, even though near-complete follow-up in these countries are noted.

		ease or diabetes, and cancer) defined by diagnoses before the start of follow-up.		considered reasonable.						
**Krug 2021**	High	No adjustment for potential confounders is reported. Analyses were stratified by age, sex, prior infection history, variant, and medical comorbidity status.	Low	'…reports processed from 1 January 2021 to 18 June 2021 with symptom codes for 'myocarditis', 'pericarditis', 'myopericarditis' or 'chest pain' for children aged 12–17 years.' Exclusions were considered reasonable.	Low	Vaccination status was linked to VAERS.	Low	Considering the observational nature of the study and recruitment of only those who had received the vaccine, this bias is unlikely to have been introduced.	Unclear	Due to the passive surveillance conducted in this study, it is impossible to determine the number of adolescents who received the vaccine and who had myocarditis events that were not reported.
**Lai 2022**	Unclear	Poisson regression models were used to generate age- and sex-adjusted incidence rate ratios. All demographic characteristics were not adjusted for in the model estimating incidence rate ratios, residual confounding cannot be completely excluded.	Low	Vaccination records and linked health records of adolescents were provided by the Department of Health.	Low	Vaccination status was checked using an electronic database system.	Low	Given the electronic vaccination records this study is based on, it is unlikely that adolescents receiving or not receiving the vaccine would not have been classified as such.	Low	Retrospective cohort study with a very short follow-up of 28 days. Likelihood of missing critical clinical information is minimal.
**Li 2022**	High	No adjustment for potential confounders is reported.	Low	Vaccination records and linked electronic health records of adolescents. The exclusion of adolescents with a history of myocarditis was considered reasonable.	Unclear	Vaccination status was checked using vaccination records. No further information is available.	Unclear	Vaccination status was determined through vaccination records. Bias due to deviations from intended interventions could not be ruled out.	Unclear	Due to the passive surveillance conducted in this study, it is impossible to determine the number of adolescents who received the vaccine and who had myocarditis events that were not reported.
**Mevorach 2022**	High	No adjustment for potential confounders is	Low	'During the period under study, 404,407 adolescents (195,579 of whom were male)	Unclear	It is not reported how vaccination status was verified.	Unclear	It is not clear how vaccination status was verified, therefore bias due to	Unclear	Due to the passive surveillance conducted in this study, it is impossible to determine

		reported. Results are stratified by sex.		received the first dose of vaccine, 326,463 adolescents (157,153 of whom were male) received the second dose, and 18 cases of myocarditis leading to hospitalization were reported.’ Exclusions due to reasonable alternative diagnoses were considered reasonable.				deviations from intended interventions could not be ruled out.		the number of adolescents who received the vaccine and who had myocarditis events that were not reported. ‘…we acquired only reports of cases of myocarditis that led to hospitalization.’
**Nygaard 2022**	High	No adjustment for potential confounders is reported. Results are stratified by sex.	Low	‘…all individuals 12–17 years of age hospitalized due to myocarditis and pericarditis after COVID-19 mRNA vaccination in the period May 15 to September 15, 2021. The setting was a multicentre study including all 18 Danish Paediatric Departments…’	Low	Vaccination status was linked to the Danish VAERS.	Low	Considering the observational nature of the study and recruitment of only those who had received the vaccine, this bias is unlikely to have been introduced.	Unclear	Due to the passive surveillance conducted in this study, it is impossible to determine the number of adolescents who received the vaccine and who had myocarditis events that were not reported. ‘…underreporting is one of the main limitations of this passive surveillance system.’
**Oster 2022**	High	No adjustment for potential confounders is reported. Results are reported by race and ethnicity, where available. Age- and sex-specific rates were calculated.	Low	‘…myocarditis to the Vaccine Adverse Event Reporting System (VAERS) that occurred after mRNA-based COVID-19 vaccine administration…’ Exclusions of cases with previous myocarditis were considered reasonable.	Low	Vaccination status was linked to VAERS.	Low	Considering the observational nature of the study and recruitment of only those who had received the vaccine, this bias is unlikely to have been introduced.	Unclear	Due to the passive surveillance conducted in this study, it is impossible to determine the number of adolescents who received the vaccine and who had myocarditis events that were not reported. ‘As such, the reports of myocarditis to VAERS may be incomplete, and

										the quality of the information reported is variable.’ ‘No assumptions or imputations were made regarding missing data.’
**Yap 2022**	High	No adjustment for potential confounders is reported. Results are reported by age and sex.	Low	‘Confirmed and probable cases based on the above definition were included. This study is a retrospective review of HAS vaccine adverse event reporting system up to 25 July 2021.’	Low	Vaccination status was linked to the Health Sciences Authority vaccine adverse event reporting system.	Low	Considering the observational nature of the study and recruitment of only those who had received the vaccine, this bias is unlikely to have been introduced.	Unclear	Due to the passive surveillance conducted in this study, it is impossible to determine the number of adolescents who received the vaccine and who had myocarditis events that were not reported.

Supplementary Table 3: Risk of bias of included studies assessed with ROBINS-I (domains 6-9)

Study	Bias in measurement of outcomes		Bias in selection of the reported result		Other source of bias		Overall bias	
	Judgment	Comment	Judgment	Comment	Judgment	Comment	Judgment	Comment
**Block 2022**	High	'…case definitions for myocarditis, pericarditis, or MIS were ICD-10-CM code–based; diagnoses were not confirmed with chart review and are subject to misclassification.'	Unclear	We did not have access to the protocol, therefore selective reporting could not be assessed.	Unclear	'…outcomes were rare in some cohorts, leading to wide CIs around RR estimates.' '…some overlap might have occurred in risk windows for persons who had a SARS-CoV-2 infection soon after vaccination or a vaccination soon after infection.' It is not clear how generalisable results from the PCORnet cohorts are to other settings.	High	Measurement of outcomes was subject to misclassification. Confounding, intervention misclassification, attrition, selective outcome reporting, threats to external validity and imprecision due to rare outcomes could not be ruled out.
**June Choe 2021**	Unclear	Some self-reporting of adverse events; the influence of self-reporting bias could not be quantified. 'Anyone, including vaccine recipients and health care providers, were encouraged to report a suspected adverse event following immunization to the government.'	Unclear	We did not have access to the protocol, therefore selective reporting could not be assessed.	Unclear	The sample is restricted to Korean high school students; therefore, the results may be limited in their generalisability.	High	Data were unadjusted and therefore subject to confounding. Passive surveillance likely resulted in selection and attrition bias. Self-reporting bias, selective outcome reporting and threats to external validity could not be ruled out.
**Chouchana 2022**	Unclear	'Another limitation is the quality of the data reported in pharmacovigilance databases, that could result in some cases in misdiagnosis.' 'On one hand, myocarditis has suffered from a notoriety bias after media communication.'	Unclear	We did not have access to the protocol, therefore selective reporting could not be assessed.	Low	None identified.	Unclear	Confounding, attrition, ascertainment of outcomes and selective outcome reporting could not be ruled out.
**Chua 2021**	Unclear	'Some patients had negative MRI results because not all MRI suites in Hong Kong's public	Unclear	We did not have access to the protocol, therefore selective reporting could not be assessed.	Unclear	The sample is restricted to adolescent in Hong Kong; therefore, the results may be limited in their generalisability.	High	Data were unadjusted and therefore subject to confounding. Attrition, ascertainment of outcomes, selective outcome reporting

		hospitals have the capability…to meet the 2018 Lake Louise Criteria for the diagnosis of myocarditis. Furthermore, the incidence of acute myocarditis/ pericarditis following the COVID-19 vaccination remained to be high, possibly attributed to increased awareness…' '…different criteria were likely used by clinicians in generating a diagnostic code among the nonvaccinated individuals for the calculation of the background myocarditis/ pericarditis incidence as it was in a non-research setting.'						and threats to external validity could not be ruled out.
**Dagan 2021**	Unclear	'…some diagnoses that were recorded in out-of-network hospitals, which were delayed in being reported to the insurer and were not entered by the person's general practitioner from the hospital discharge notes into the outpatient medical record, could have been missed. Fourth, it is possible that persons are more likely to increase their levels of clinical awareness, concern, or both after vaccination or SARS-CoV-2 infection…'	Unclear	We did not have access to the protocol, therefore selective reporting could not be assessed.	Unclear	Generalisability is limited due to the inclusion of only participants with health insurance within Clalit Health Services. 'Because this different composition changes the population over which the causal effect is being estimated, different estimates might be found for adverse events for which the incidence may differ substantially between subgroups (e.g., myocarditis). Also, we excluded certain populations…that could be at particularly high risk for certain adverse events. Both issues should be taken into account when considering the generalizability of the findings.'	Unclear	Confounding, attrition, ascertainment of outcomes, selective outcome reporting and threats to external validity could not be ruled out.
**Das 2021**	Low	Physician-assessed '…probable myopericarditis as per the CDC-defined criteria for	Unclear	We did not have access to the protocol, therefore selective	Unclear	Generalisability is limited by the small number of participants from the US only.	High	Data were unadjusted and therefore subject to confounding. Bias in

		diagnosis of myocarditis and pericarditis.’		reporting could not be assessed.				classification of the intervention and due to deviations from intended interventions, attrition, selective outcome reporting and threats to external validity could not be ruled out.
**Foltran 2022**	Unclear	It is not reported how myocarditis was defined and assessed in various countries.	Unclear	We did not have access to the protocol, therefore selective reporting could not be assessed.	Unclear	‘As the US pharmacovigilance data did not include dose information (dose 1 or dose 2), we were unable to analyse the reports. This lack of information is a potential limitation of our study on the transferability of the results to the US vaccination context and may have limited the statistical power of our study, particularly when comparing the two vaccines.’	Unclear	Bias in classification of the intervention and due to deviations from intended interventions, attrition, bias related to ascertainment of outcomes, selective outcome reporting and limitations of generalisability to the US context could not be ruled out.
**Funck 2022**	Unclear	‘Optum is a health claims database in FDA’s CBER BEST System which has inherent limitations, such as small sample sizes and imperfect sensitivity of ICD-10 codes to identify these rare outcomes. We also acknowledge that the cases were not validated by a complete review of patient’s medical records. The lack of chart review validation may lead to overestimation of myocarditis/pericarditis case rate.’	Unclear	We did not have access to the protocol, therefore selective reporting could not be assessed.	Unclear	‘Our analysis was conducted before the emergence of the Omicron variant. The estimated benefits of the vaccine would decrease if the vaccine became less effective against novel variants of COVID-19, but benefit would increase if COVID-19 incidence increased. The durability of vaccine protection is another source of uncertainty for the model.’ ‘…VSD has a smaller database than the BEST system and its generalizability is limited to patients who have health maintenance organization (HMO) insurance with a high emphasis on wellness/ preventative care.’	High	There is a high risk of bias due to potential confounding. Attrition, bias related to ascertainment of outcomes, selective outcome reporting, threats to external validity of some data sources and limitations in the evidence base for variants of concern could not be ruled out.
**Gurdasani 2021**	Unclear	It is not reported how myocarditis was defined and assessed in various settings.	Unclear	We did not have access to the protocol, therefore selective reporting could not be assessed.	Unclear	‘We do not assess risks separately for one dose and two doses of vaccines...’	High	Data were unadjusted and therefore subject to confounding. Bias in classification of the intervention and due to

								deviations from intended interventions, attrition, bias in measurement of outcomes, selective outcome reporting and uncertainty around the effect of single doses could not be ruled out.
**Hause 2021**	Unclear	The authors report that '…VAERS is a passive surveillance system and is subject to underreporting and reporting biases…health care providers are required to report all serious events following vaccination. Second, medical review of reported deaths following vaccination is dependent on availability of medical records, death certificates, and autopsy reports, which might be unavailable or not available in a timely manner. Third, lack of a statistical safety signal in planned monitoring does not preclude a safety concern… Fourth, this study was not designed to identify all cases of myocarditis; only reports that listed the MedDRA term "myocarditis" were included.'	Unclear	We did not have access to the protocol, therefore selective reporting could not be assessed.	Unclear	'…v-safe is a voluntary self-enrolment program that requires children aged <15 years be enrolled by a parent or guardian and relies on vaccine administrators to promote the program. Therefore, v-safe data might not be generalizable to the overall vaccinated adolescent population.' Given the heterogeneous composition of the American population, generalisability with regards to ethnicity is less limited.	High	There is a high risk of bias due to potential confounding. Attrition and ascertainment biases, selection bias and limitations to generalisability could also not be ruled out.
**Hause 2022**	Unclear	'…as a passive surveillance system, VAERS is subject to reporting biases and underreporting, especially of nonserious events.' '…it is possible that vaccinees who experience an adverse event could be more likely to	Unclear	We did not have access to the protocol, therefore selective reporting could not be assessed.	Unclear	'…v-safe is a voluntary program; therefore, data might not be representative of the vaccinated population.' Given the heterogeneous composition of the American population, generalisability with regards to ethnicity is less limited.	Unclear	Attrition and ascertainment biases, selection bias and limitations to generalisability could not be ruled out. Myocarditis counts are subject to change.

		respond to v-safe surveys… Finally, assessment of myocarditis reports to VAERS is ongoing, and counts are subject to change.'						
**Jain 2021**	Unclear	'The 2009 Lake Louise criteria or, when parametric mapping was available, the 2018 revised Lake Louise CMR criteria were used to test for a diagnosis of acute myocarditis. ' '…combining patients from 16 different institutions that used similar but not identical approaches during the workup and management of childhood myocarditis.'	Unclear	We did not have access to the protocol, therefore selective reporting could not be assessed.	Unclear	'Our patients represent a selected cohort that may not necessarily be representative of the general paediatric population.'	High	There is a high risk of bias due to potential confounding. Attrition, bias in measurement of the outcomes, selective outcome reporting and threats to external validity could not be ruled out.
**Karlstad 2022**	Unclear	Outcomes are explicitly defined in eTable 2. '…without access to data on clinical measures, such as troponin levels, diagnostic imaging results, and endomyo-cardial biopsy, we studied myocarditis as diagnosed in clinical practice and could therefore not assess how many of these patients fulfilled all criteria for receiving a myocarditis diagnosis.'	Unclear	We did not have access to the protocol, therefore selective reporting could not be assessed.	Unclear	'Our findings in children aged 12 to 15 years were limited to relatively few exposed individuals because vaccination in this age group only recently started in most countries.' 'Surveillance bias, whereby increased focus and media attention on myocarditis as an adverse event after vaccination resulted in more subclinical cases being diagnosed, cannot be ruled out.'	Unclear	Attrition, bias in the measurement of outcomes, selective outcome reporting, limitations of sample size and surveillance bias could not be ruled out
**Krug 2021**	Low	'Reports were required to meet the CDC working definition for probable acute myocarditis…' 'A concern about a passive reporting system such as VAERS is the risk of over-ascertainment. To address this concern, we aligned our inclusion criteria with the	Unclear	We did not have access to the protocol, therefore selective reporting could not be assessed.	Unclear	'In our sample, approximately 15% of cases had an unknown dose number, similar to the CDC's reports. We allocated these cases using the proportion of reports with known dose number.' 'Our analysis only describes rates associated with the Pfizer-BioNTech vaccine.' 'Our sample only includes 23	High	There is a high risk of bias due to potential confounding. Attrition, selective outcome reporting, uncertainty regarding the effect of number of doses and limitations related to generalisability for type of vaccine and events in girls could not be ruled out.

		CDC's case definition…'				cases in girls, which is a limitation of this data set…'		
**Lai 2022**	Low	Objective measurement was used from the list of 30 AEFIs using the International Classification of Diseases, Ninth Revision (ICD-9) and International Classification of Primary Care (ICPC).	Unclear	We did not have access to the protocol, therefore selective reporting could not be assessed.	Unclear	The authors acknowledge that adolescents may have sought medical care for AEs in the private sector, therefor some AEs may not have been detected. Diagnostic codes were used as proxies for disease. The study population is predominantly Chinese, and generalisability is unclear.	Unclear	Residual confounding and selective outcome reporting could not be ruled out. Some outcomes may have been missed due to the way in which participants sought care; this is expected to affect vaccinated and unvaccinated participants equally. Some misclassification is possible, and generalisability may be limited.
**Li 2022**	Low	'…inpatient myocarditis cases were identified using the International Classification of Diseases, Ninth Revision, Clinical Modification.'	Unclear	We did not have access to the protocol, therefore selective reporting could not be assessed.	Unclear	'Limitations include sample size during the post policy period.' Generalisability is limited to adolescents in Hong Kong, and data were generated before the arrival of the Omicron variant.	High	Data were unadjusted and therefore subject to confounding. Bias in classification of the intervention and due to deviations from intended interventions, attrition, selective outcome reporting, sample size limitations, threats to external validity and a lack of information for the Omicron variant could not be ruled out.
**Mevorach 2022**	Unclear	'Clinical data that involved International Classification of Diseases, 10th Revision, 422.0-9x and 429.0x codes were reviewed by a cardiologist and a rheumatologist, and the severity of disease was classified according to the Brighton Collaboration Case Definition for myocarditis.' 'Limitations of the current study are that myocarditis was not validated on myocardial biopsy, that misclassification and reporting bias may have	Unclear	We did not have access to the protocol, therefore selective reporting could not be assessed.	Low	None identified.	High	Data were unadjusted and therefore subject to confounding. Bias in classification of the intervention and due to deviations from intended interventions, attrition, bias in measurement of outcomes and selective outcome reporting could not be ruled out.

		taken place…'						
**Nygaard 2022**	Unclear	'Further, our incidences may be overestimated due to possible inclusion of cases unrelated to the vaccine, but occurring in vaccinated adolescents…On the contrary, our incidences may also be underestimated.'	Unclear	We did not have access to the protocol, therefore selective reporting could not be assessed.	Low	None identified.	High	Data were unadjusted and therefore subject to confounding. Attrition, bias in measurement of outcomes and selective outcome reporting could not be ruled out.
**Oster 2022**	Unclear	'Furthermore, as a passive system, VAERS data are subject to reporting biases in that both underreporting and overreporting are possible. Given the high verification rate of reports of myocarditis to VAERS after mRNA-based COVID-19 vaccination, underreporting is more likely. Therefore, the actual rates of myocarditis per million doses of vaccine is likely higher than estimated.'	Unclear	We did not have access to the protocol, therefore selective reporting could not be assessed.	Unclear	'…the data from vaccination administration were limited to what is reported to the CDC and thus may be incomplete, particularly about demographics.'	High	Data were unadjusted and therefore subject to confounding. Attrition, bias in measurement of outcomes, selective outcome reporting and limitations around vaccination administration data could not be ruled out.
**Yap 2022**	Low	'Reports of myocarditis and pericarditis are reviewed and adjudicated using the US Centres for Disease Control and Prevention (CDC) definition of myocarditis and pericarditis by an independent expert panel of cardiologists.'	Unclear	We did not have access to the protocol, therefore selective reporting could not be assessed.	Low	None identified.	High	Data were unadjusted and therefore subject to confounding. Attrition and selective outcome reporting could not be ruled out.

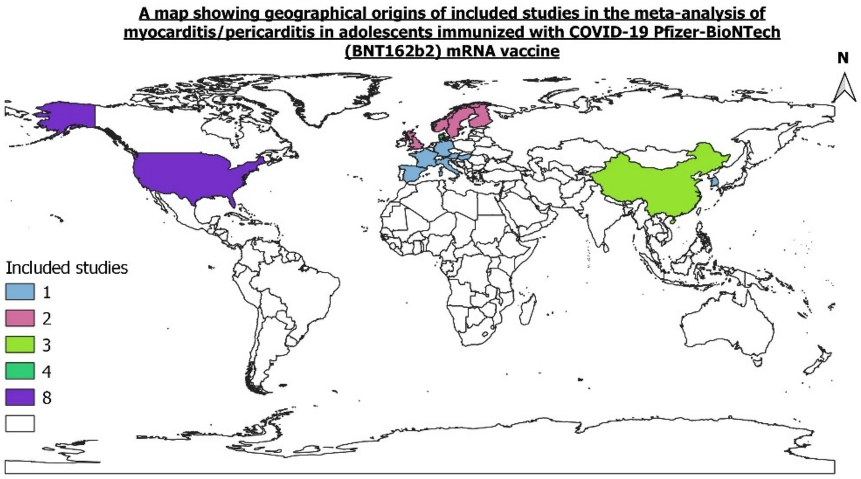

Supplementary Figure 1: Map showing geographical origins of included studies

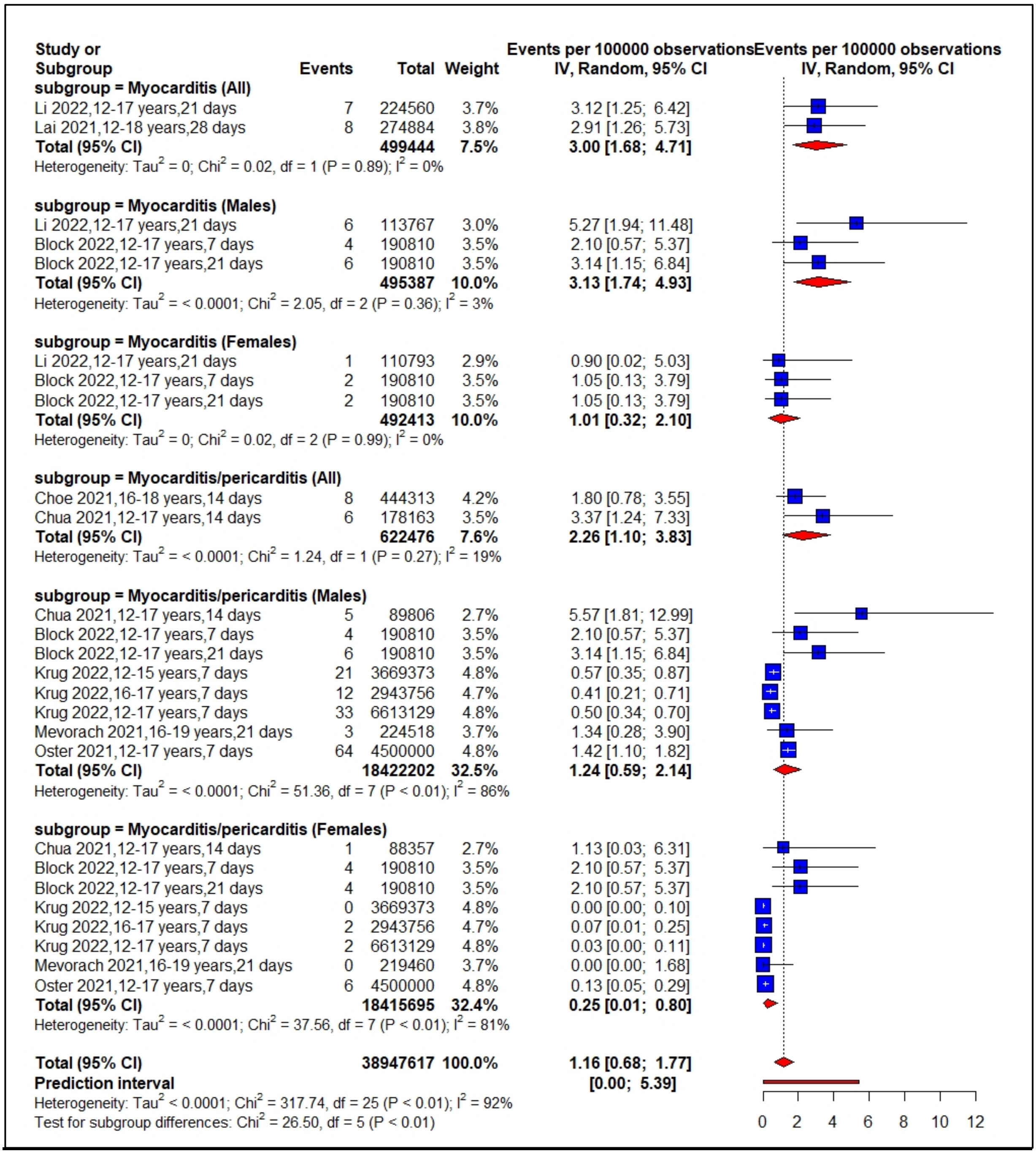

Supplementary Figure 2: **Pooled proportion of myocarditis or of myocarditis/pericarditis following the first dose of BNT162b2 vaccination against COVID-19 among adolescents**. Subgroup are presented to consider sex. The pooled incidence of studied inflammatory heart conditions following the first dose was 1.16 cases per 100,000 doses of BNT162b2 vaccine. The whisker represents the 95% confidence interval. The inclusion period of participants spanned from December 2020 to February 2022. Days indicate the period length after BNT162b2 vaccination used by the study. E.g.: 7 days will read as 0-7 days post BNT162b2 vaccination.

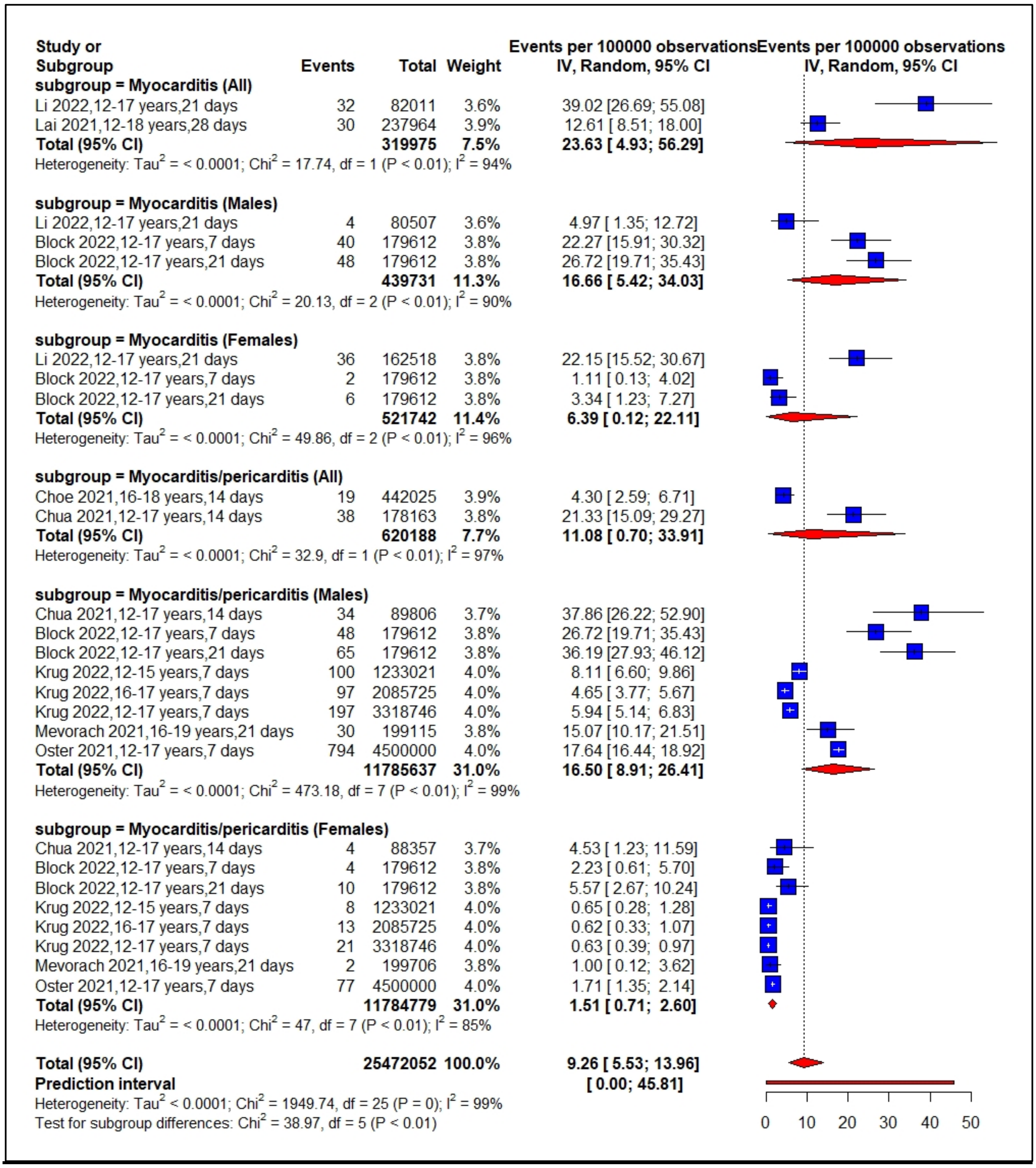

Supplementary Figure 3: **Pooled proportion of myocarditis or of myocarditis/pericarditis following the second dose of BNT162b2 vaccination against COVID-19 among adolescents**. Subgroup are presented to consider sex. The pooled incidence of studied inflammatory heart conditions following the second dose was 9.26 cases per 100,000 doses of BNT162b2 vaccine. The whisker represents the 95% confidence interval. The inclusion period of participants spanned from December 2020 to February 2022. Days indicate the period length after BNT162b2 vaccination used by the study. E.g.: 7 days will read as 0-7 days post BNT162b2 vaccination.

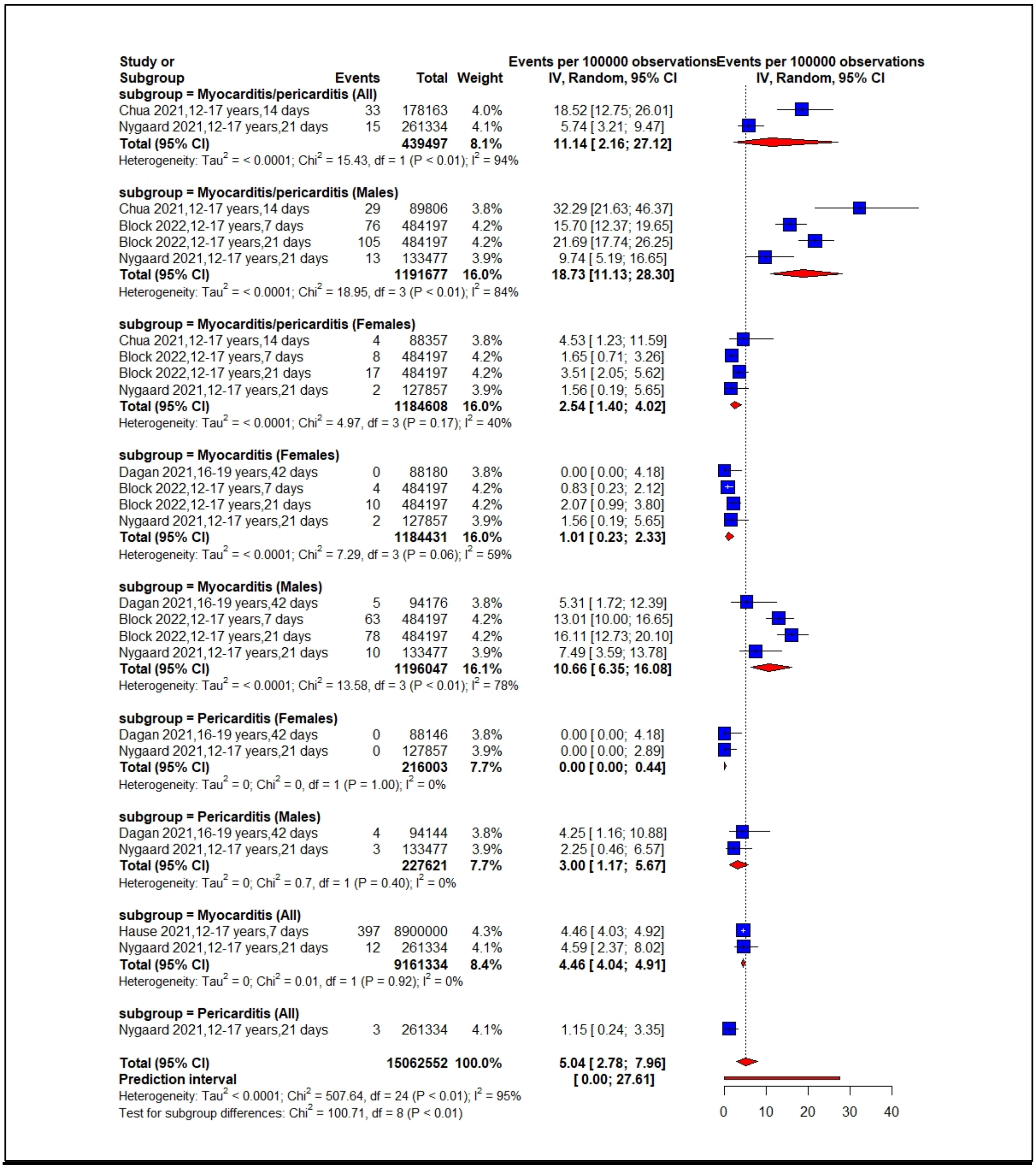

Supplementary Figure 4: **Pooled proportion of myocarditis or pericarditis or of myocarditis/pericarditis following any dose of BNT162b2 vaccination against COVID-19 among adolescents**. Subgroup are presented to consider sex. The pooled incidence of studied inflammatory heart conditions following any dose was 5.04 cases per 100,000 doses of BNT162b2 vaccine. The whisker represents the 95% confidence interval. The inclusion period of participants spanned from December 2020 to February 2022. Days indicate the period length after BNT162b2 vaccination used by the study. E.g.: 7 days will read as 0-7 days post BNT162b2 vaccination.

Study or Subgroup	Events	Total	Weight	Events per 100000 observations IV, Random, 95% CI
**subgroup = Myocarditis (All)**				
Li 2022,12-17 years,Dose 1,21 days	7	224560	1.3%	3.12 [1.25; 6.42]
Li 2022,12-17 years,Dose 2,21 days	32	82011	1.2%	39.02 [26.69; 55.08]
Hause 2021,12-17 years,Any dose,7 days	397	8900000	1.3%	4.46 [4.03; 4.92]
Hause 2022,12-17 years,Booster dose,7 days	32	2800000	1.3%	1.14 [0.78; 1.61]
Lai 2021,12-18 years,Dose 1,28 days	8	274884	1.3%	2.91 [1.26; 5.73]
Lai 2021,12-18 years,Dose 2,28 days	30	237964	1.3%	12.61 [8.51; 18.00]
Nygaard 2021,12-17 years,Any dose,21 days	12	261334	1.3%	4.59 [2.37; 8.02]
**Total (95% CI)**		**12780753**	**9.0%**	**6.82 [1.86; 14.90]**
Heterogeneity: Tau^2 = < 0.0001; Chi^2 = 182.19, df = 6 ($P < 0.01$); I^2 = 97%				
**subgroup = Myocarditis (Males)**				
Li 2022,12-17 years,Dose 1,21 days	6	113767	1.2%	5.27 [1.94; 11.48]
Li 2022,12-17 years,Dose 2,21 days	4	80507	1.2%	4.97 [1.35; 12.72]
Dagan 2021,16-19 years,Any dose,42 days	5	94176	1.2%	5.31 [1.72; 12.39]
Block 2022,12-17 years,Dose 1,7 days	4	190810	1.3%	2.10 [0.57; 5.37]
Block 2022,12-17 years,Dose 1,21 days	6	190810	1.3%	3.14 [1.15; 6.84]
Block 2022,12-17 years,Dose 2,7 days	40	179612	1.3%	22.27 [15.91; 30.32]
Block 2022,12-17 years,Dose 2,21 days	48	179612	1.3%	26.72 [19.71; 35.43]
Block 2022,12-17 years,Any dose,7 days	63	484197	1.3%	13.01 [10.00; 16.65]
Block 2022,12-17 years,Any dose,21 days	78	484197	1.3%	16.11 [12.73; 20.10]
Nygaard 2021,12-17 years,Any dose,21 days	10	133477	1.2%	7.49 [3.59; 13.78]
**Total (95% CI)**		**2131165**	**12.6%**	**9.33 [5.09; 14.84]**
Heterogeneity: Tau^2 = < 0.0001; Chi^2 = 108.86, df = 9 ($P < 0.01$); I^2 = 92%				
**subgroup = Myocarditis (Females)**				
Li 2022,12-17 years,Dose 1,21 days	1	110793	1.2%	0.90 [0.02; 5.03]
Li 2022,12-17 years,Dose 2,21 days	36	162518	1.3%	22.15 [15.52; 30.67]
Dagan 2021,16-19 years,Any dose,42 days	0	88180	1.2%	0.00 [0.00; 4.18]
Block 2022,12-17 years,Dose 1,7 days	2	190810	1.3%	1.05 [0.13; 3.79]
Block 2022,12-17 years,Dose 1,21 days	2	190810	1.3%	1.05 [0.13; 3.79]
Block 2022,12-17 years,Dose 2,7 days	2	179612	1.3%	1.11 [0.13; 4.02]
Block 2022,12-17 years,Dose 2,21 days	6	179612	1.3%	3.34 [1.23; 7.27]
Block 2022,12-17 years,Any dose,7 days	4	484197	1.3%	0.83 [0.23; 2.12]
Block 2022,12-17 years,Any dose,21 days	10	484197	1.3%	2.07 [0.99; 3.80]
Nygaard 2021,12-17 years,Any dose,21 days	2	127857	1.2%	1.56 [0.19; 5.65]
**Total (95% CI)**		**2198586**	**12.7%**	**2.04 [0.44; 4.79]**
Heterogeneity: Tau^2 = < 0.0001; Chi^2 = 88.03, df = 9 ($P < 0.01$); I^2 = 90%				
**subgroup = Myocarditis/pericarditis (All)**				
Choe 2021,16-18 years,Dose 1,14 days	8	444313	1.3%	1.80 [0.78; 3.55]
Choe 2021,16-18 years,Dose 2,14 days	19	442025	1.3%	4.30 [2.59; 6.71]
Chua 2021,12-17 years,Any dose,14 days	33	178163	1.3%	18.52 [12.75; 26.01]
Chua 2021,12-17 years,Dose 1,14 days	6	178163	1.3%	3.37 [1.24; 7.33]
Chua 2021,12-17 years,Dose 2,14 days	38	178163	1.3%	21.33 [15.09; 29.27]
Nygaard 2021,12-17 years,Any dose,21 days	15	261334	1.3%	5.74 [3.21; 9.47]
**Total (95% CI)**		**1682161**	**7.7%**	**7.53 [2.75; 14.66]**
Heterogeneity: Tau^2 = < 0.0001; Chi^2 = 84.96, df = 5 ($P < 0.01$); I^2 = 94%				
**subgroup = Myocarditis/pericarditis (Males)**				
Chua 2021,12-17 years,Any dose,14 days	29	89806	1.2%	32.29 [21.63; 46.37]
Chua 2021,12-17 years,Dose 1,14 days	5	89806	1.2%	5.57 [1.81; 12.99]
Chua 2021,12-17 years,Dose 2,14 days	34	89806	1.2%	37.86 [26.22; 52.90]
Block 2022,12-17 years,Dose 1,7 days	4	190810	1.3%	2.10 [0.57; 5.37]
Block 2022,12-17 years,Dose 1,21 days	6	190810	1.3%	3.14 [1.15; 6.84]
Block 2022,12-17 years,Dose 2,7 days	48	179612	1.3%	26.72 [19.71; 35.43]
Block 2022,12-17 years,Dose 2,21 days	65	179612	1.3%	36.19 [27.93; 46.12]
Block 2022,12-17 years,Any dose,7 days	76	484197	1.3%	15.70 [12.37; 19.65]
Block 2022,12-17 years,Any dose,21 days	105	484197	1.3%	21.69 [17.74; 26.25]
Krug 2022,12-15 years,Dose 1,7 days	21	3669373	1.3%	0.57 [0.35; 0.87]
Krug 2022,12-15 years,Dose 2,7 days	100	1233021	1.3%	8.11 [6.60; 9.86]
Krug 2022,16-17 years,Dose 1,7 days	12	2943756	1.3%	0.41 [0.21; 0.71]
Krug 2022,16-17 years,Dose 2,7 days	97	2085725	1.3%	4.65 [3.77; 5.67]
Krug 2022,12-17 years,Dose 1,7 days	33	6613129	1.3%	0.50 [0.34; 0.70]
Krug 2022,12-17 years,Dose 2,7 days	197	3318746	1.3%	5.94 [5.14; 6.83]
Mevorach 2021,16-19 years,Dose 1,21 days	3	224518	1.3%	1.34 [0.28; 3.90]
Mevorach 2021,16-19 years,Dose 2,21 days	30	199115	1.3%	15.07 [10.17; 21.51]
Nygaard 2021,12-17 years,Any dose,21 days	13	133477	1.2%	9.74 [5.19; 16.65]
Oster 2021,12-17 years,Dose 1,7 days	64	4500000	1.3%	1.42 [1.10; 1.82]
Oster 2021,12-17 years,Dose 2,7 days	794	4500000	1.3%	17.64 [16.44; 18.92]
**Total (95% CI)**		**31399516**	**25.9%**	**8.94 [4.78; 14.39]**
Heterogeneity: Tau^2 = < 0.0001; Chi^2 = 2477.87, df = 19 ($P = 0$); I^2 = 99%				
**subgroup = Myocarditis/pericarditis (Females)**				
Chua 2021,12-17 years,Any dose,14 days	4	88357	1.2%	4.53 [1.23; 11.59]
Chua 2021,12-17 years,Dose 1,14 days	1	88357	1.2%	1.13 [0.03; 6.31]
Chua 2021,12-17 years,Dose 2,14 days	4	88357	1.2%	4.53 [1.23; 11.59]
Block 2022,12-17 years,Dose 1,7 days	4	190810	1.3%	2.10 [0.57; 5.37]
Block 2022,12-17 years,Dose 1,21 days	4	190810	1.3%	2.10 [0.57; 5.37]
Block 2022,12-17 years,Dose 2,7 days	4	179612	1.3%	2.23 [0.61; 5.70]
Block 2022,12-17 years,Dose 2,21 days	10	179612	1.3%	5.57 [2.67; 10.24]
Block 2022,12-17 years,Any dose,7 days	8	484197	1.3%	1.65 [0.71; 3.26]
Block 2022,12-17 years,Any dose,21 days	17	484197	1.3%	3.51 [2.05; 5.62]
Krug 2022,12-15 years,Dose 1,7 days	0	3669373	1.3%	0.00 [0.00; 0.10]
Krug 2022,12-15 years,Dose 2,7 days	8	1233021	1.3%	0.65 [0.28; 1.28]
Krug 2022,16-17 years,Dose 1,7 days	2	2943756	1.3%	0.07 [0.01; 0.25]
Krug 2022,16-17 years,Dose 2,7 days	13	2085725	1.3%	0.62 [0.33; 1.07]
Krug 2022,12-17 years,Dose 1,7 days	2	6613129	1.3%	0.03 [0.00; 0.11]
Krug 2022,12-17 years,Dose 2,7 days	21	3318746	1.3%	0.63 [0.39; 0.97]
Mevorach 2021,16-19 years,Dose 1,21 days	0	219460	1.3%	0.00 [0.00; 1.68]
Mevorach 2021,16-19 years,Dose 2,21 days	2	199706	1.3%	1.00 [0.12; 3.62]
Nygaard 2021,12-17 years,Any dose,21 days	2	127857	1.2%	1.56 [0.19; 5.65]
Oster 2021,12-17 years,Dose 1,7 days	6	4500000	1.3%	0.13 [0.05; 0.29]
Oster 2021,12-17 years,Dose 2,7 days	77	4500000	1.3%	1.71 [1.35; 2.14]
**Total (95% CI)**		**31385082**	**25.9%**	**1.05 [0.52; 1.77]**
Heterogeneity: Tau^2 = < 0.0001; Chi^2 = 327.76, df = 19 ($P < 0.01$); I^2 = 94%				
**subgroup = Pericarditis (Females)**				
Dagan 2021,16-19 years,Any dose,42 days	0	88146	1.2%	0.00 [0.00; 4.18]
Nygaard 2021,12-17 years,Any dose,21 days	0	127857	1.2%	0.00 [0.00; 2.89]
**Total (95% CI)**		**216003**	**2.4%**	**0.00 [0.00; 0.44]**
Heterogeneity: Tau^2 = 0; Chi^2 = 0, df = 1 ($P = 1.00$); I^2 = 0%				
**subgroup = Pericarditis (Males)**				
Dagan 2021,16-19 years,Any dose,42 days	4	94144	1.2%	4.25 [1.16; 10.88]
Nygaard 2021,12-17 years,Any dose,21 days	3	133477	1.2%	2.25 [0.46; 6.57]
**Total (95% CI)**		**227621**	**2.5%**	**3.00 [1.17; 5.67]**
Heterogeneity: Tau^2 = 0; Chi^2 = 0.7, df = 1 ($P = 0.40$); I^2 = 0%				
**subgroup = Pericarditis (All)**				
Nygaard 2021,12-17 years,Any dose,21 days	3	261334	1.3%	1.15 [0.24; 3.35]
**Total (95% CI)**		**82282221**	**100.0%**	**4.50 [3.14; 6.11]**
**Prediction interval**				**[0.00; 27.12]**
Heterogeneity: Tau^2 < 0.0001; Chi^2 = 4947.07, df = 77 ($P = 0$); I^2 = 98%				
Test for subgroup differences: Chi^2 = 65.94, df = 8 ($P < 0.01$)				

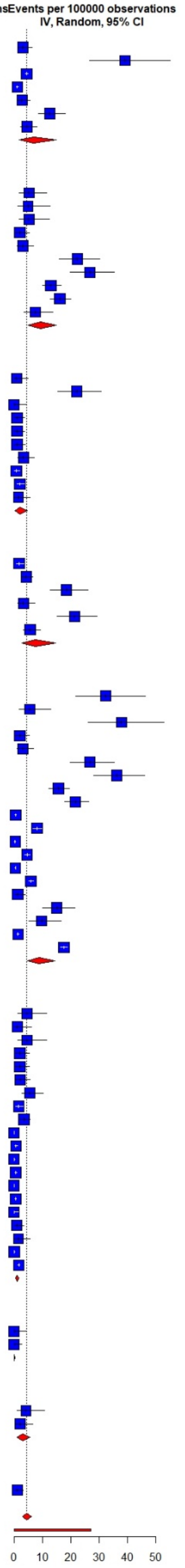

Supplementary Figure 5: **Pooled proportion of myocarditis or pericarditis or of myocarditis/pericarditis across all doses including booster dose of BNT162b2 vaccination against COVID-19 among adolescents.** Subgroup are presented to consider sex. The pooled incidence of studied inflammatory heart conditions across all doses was 4.50 cases per 100,000 doses of BNT162b2 vaccine. The whisker represents the 95% confidence interval. The inclusion period of participants spanned from December 2020 to February 2022. Days indicate the period length after BNT162b2 vaccination used by the study. E.g.: 7 days will read as 0-7 days post BNT162b2 vaccination.

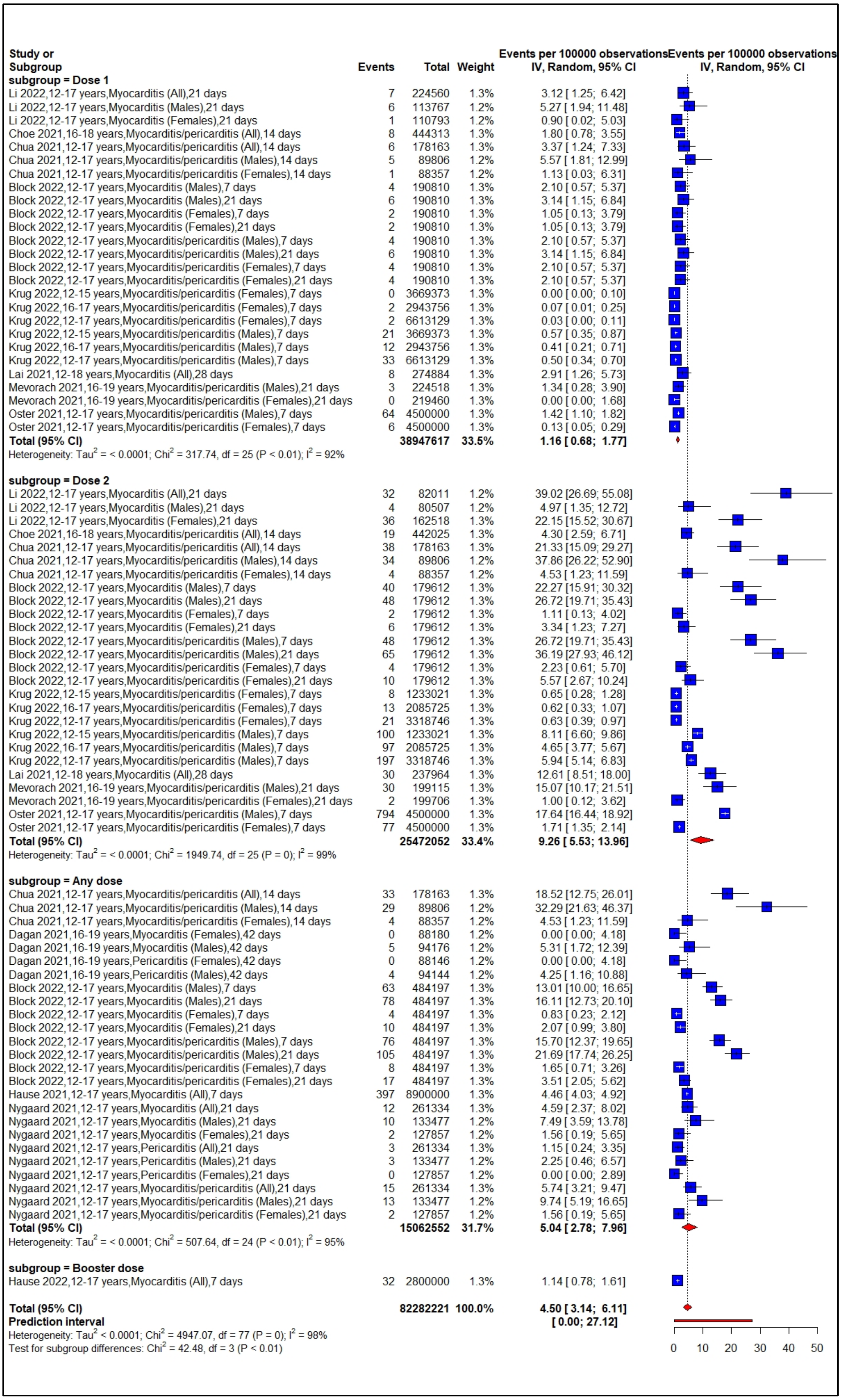

Supplementary Figure 6: Pooled proportion of myocarditis or pericarditis or of myocarditis/pericarditis stratified by dose of BNT162b2 vaccination against COVID-19 among adolescents. The whisker represents the 95% confidence interval. The inclusion period of participants spanned from December 2020 to February 2022

Study or Subgroup	Events	Total	Weight	Events per 100000 observations IV, Random, 95% CI
**subgroup = Myocarditis (All)**				
Li 2022,12-17 years,Dose 1,21 days	7	224560	1.9%	3.12 [1.25; 6.42]
Li 2022,12-17 years,Dose 2,21 days	32	82011	1.8%	39.02 [26.69; 55.08]
Lai 2021,12-18 years,Dose 1,28 days	8	274884	1.9%	2.91 [1.26; 5.73]
Lai 2021,12-18 years,Dose 2,28 days	30	237964	1.9%	12.61 [8.51; 18.00]
**Total (95% CI)**		**819419**	**7.6%**	**10.78 [1.51; 28.50]**
Heterogeneity: Tau^2 = < 0.0001; Chi^2 = 66.86, df = 3 (P < 0.01); I^2 = 96%				
**subgroup = Myocarditis (Males)**				
Li 2022,12-17 years,Dose 1,21 days	6	113767	1.8%	5.27 [1.94; 11.48]
Li 2022,12-17 years,Dose 2,21 days	4	80507	1.8%	4.97 [1.35; 12.72]
Block 2022,12-17 years,Dose 1,7 days	4	190810	1.9%	2.10 [0.57; 5.37]
Block 2022,12-17 years,Dose 1,21 days	6	190810	1.9%	3.14 [1.15; 6.84]
Block 2022,12-17 years,Dose 2,7 days	40	179612	1.9%	22.27 [15.91; 30.32]
Block 2022,12-17 years,Dose 2,21 days	48	179612	1.9%	26.72 [19.71; 35.43]
**Total (95% CI)**		**935118**	**11.3%**	**8.69 [2.76; 17.95]**
Heterogeneity: Tau^2 = < 0.0001; Chi^2 = 89.07, df = 5 (P < 0.01); I^2 = 94%				
**subgroup = Myocarditis (Females)**				
Li 2022,12-17 years,Dose 1,21 days	1	110793	1.8%	0.90 [0.02; 5.03]
Li 2022,12-17 years,Dose 2,21 days	36	162518	1.9%	22.15 [15.52; 30.67]
Block 2022,12-17 years,Dose 1,7 days	2	190810	1.9%	1.05 [0.13; 3.79]
Block 2022,12-17 years,Dose 1,21 days	2	190810	1.9%	1.05 [0.13; 3.79]
Block 2022,12-17 years,Dose 2,7 days	2	179612	1.9%	1.11 [0.13; 4.02]
Block 2022,12-17 years,Dose 2,21 days	6	179612	1.9%	3.34 [1.23; 7.27]
**Total (95% CI)**		**1014155**	**11.4%**	**3.12 [0.34; 8.69]**
Heterogeneity: Tau^2 = < 0.0001; Chi^2 = 71.23, df = 5 (P < 0.01); I^2 = 93%				
**subgroup = Myocarditis/pericarditis (All)**				
Choe 2021,16-18 years,Dose 1,14 days	8	444313	2.0%	1.80 [0.78; 3.55]
Choe 2021,16-18 years,Dose 2,14 days	19	442025	2.0%	4.30 [2.59; 6.71]
Chua 2021,12-17 years,Dose 1,14 days	6	178163	1.9%	3.37 [1.24; 7.33]
Chua 2021,12-17 years,Dose 2,14 days	38	178163	1.9%	21.33 [15.09; 29.27]
**Total (95% CI)**		**1242664**	**7.7%**	**6.01 [1.06; 15.00]**
Heterogeneity: Tau^2 = < 0.0001; Chi^2 = 55.84, df = 3 (P < 0.01); I^2 = 95%				
**subgroup = Myocarditis/pericarditis (Males)**				
Chua 2021,12-17 years,Dose 1,14 days	5	89806	1.8%	5.57 [1.81; 12.99]
Chua 2021,12-17 years,Dose 2,14 days	34	89806	1.8%	37.86 [26.22; 52.90]
Block 2022,12-17 years,Dose 1,7 days	4	190810	1.9%	2.10 [0.57; 5.37]
Block 2022,12-17 years,Dose 1,21 days	6	190810	1.9%	3.14 [1.15; 6.84]
Block 2022,12-17 years,Dose 2,7 days	48	179612	1.9%	26.72 [19.71; 35.43]
Block 2022,12-17 years,Dose 2,21 days	65	179612	1.9%	36.19 [27.93; 46.12]
Krug 2022,12-15 years,Dose 1,7 days	21	3669373	2.0%	0.57 [0.35; 0.87]
Krug 2022,12-15 years,Dose 2,7 days	100	1233021	2.0%	8.11 [6.60; 9.86]
Krug 2022,16-17 years,Dose 1,7 days	12	2943756	2.0%	0.41 [0.21; 0.71]
Krug 2022,16-17 years,Dose 2,7 days	97	2085725	2.0%	4.65 [3.77; 5.67]
Krug 2022,12-17 years,Dose 1,7 days	33	6613129	2.0%	0.50 [0.34; 0.70]
Krug 2022,12-17 years,Dose 2,7 days	197	3318746	2.0%	5.94 [5.14; 6.83]
Mevorach 2021,16-19 years,Dose 1,21 days	3	224518	1.9%	1.34 [0.28; 3.90]
Mevorach 2021,16-19 years,Dose 2,21 days	30	199115	1.9%	15.07 [10.17; 21.51]
Oster 2021,12-17 years,Dose 1,7 days	64	4500000	2.0%	1.42 [1.10; 1.82]
Oster 2021,12-17 years,Dose 2,7 days	794	4500000	2.0%	17.64 [16.44; 18.92]
**Total (95% CI)**		**30207839**	**31.0%**	**7.06 [3.07; 12.68]**
Heterogeneity: Tau^2 = < 0.0001; Chi^2 = 2174.94, df = 15 (P = 0); I^2 = 99%				
**subgroup = Myocarditis/pericarditis (Females)**				
Chua 2021,12-17 years,Dose 1,14 days	1	88357	1.8%	1.13 [0.03; 6.31]
Chua 2021,12-17 years,Dose 2,14 days	4	88357	1.8%	4.53 [1.23; 11.59]
Block 2022,12-17 years,Dose 1,7 days	4	190810	1.9%	2.10 [0.57; 5.37]
Block 2022,12-17 years,Dose 1,21 days	4	190810	1.9%	2.10 [0.57; 5.37]
Block 2022,12-17 years,Dose 2,7 days	4	179612	1.9%	2.23 [0.61; 5.70]
Block 2022,12-17 years,Dose 2,21 days	10	179612	1.9%	5.57 [2.67; 10.24]
Krug 2022,12-15 years,Dose 1,7 days	0	3669373	2.0%	0.00 [0.00; 0.10]
Krug 2022,12-15 years,Dose 2,7 days	8	1233021	2.0%	0.65 [0.28; 1.28]
Krug 2022,16-17 years,Dose 1,7 days	2	2943756	2.0%	0.07 [0.01; 0.25]
Krug 2022,16-17 years,Dose 2,7 days	13	2085725	2.0%	0.62 [0.33; 1.07]
Krug 2022,12-17 years,Dose 1,7 days	2	6613129	2.0%	0.03 [0.00; 0.11]
Krug 2022,12-17 years,Dose 2,7 days	21	3318746	2.0%	0.63 [0.39; 0.97]
Mevorach 2021,16-19 years,Dose 1,21 days	0	219460	1.9%	0.00 [0.00; 1.68]
Mevorach 2021,16-19 years,Dose 2,21 days	2	199706	1.9%	1.00 [0.12; 3.62]
Oster 2021,12-17 years,Dose 1,7 days	6	4500000	2.0%	0.13 [0.05; 0.29]
Oster 2021,12-17 years,Dose 2,7 days	77	4500000	2.0%	1.71 [1.35; 2.14]
**Total (95% CI)**		**30200474**	**31.0%**	**0.79 [0.31; 1.49]**
Heterogeneity: Tau^2 = < 0.0001; Chi^2 = 273.46, df = 15 (P < 0.01); I^2 = 95%				
**Total (95% CI)**		**64419669**	**100.0%**	**4.36 [2.69; 6.42]**
**Prediction interval**				**[0.00; 28.64]**
Heterogeneity: Tau^2 < 0.0001; Chi^2 = 3924.39, df = 51 (P = 0); I^2 = 99%				
Test for subgroup differences: Chi^2 = 27.52, df = 5 (P < 0.01)				

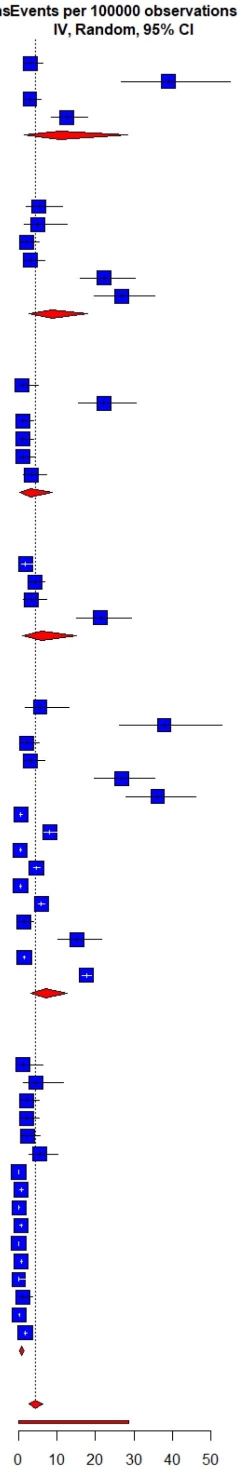

Supplementary Figure 7: Pooled proportion of myocarditis or of myocarditis/pericarditis following primary doses of BNT162b2 vaccination against COVID-19 among adolescents. The whisker represents the 95% confidence interval. The inclusion period of participants spanned from December 2020 to February 2022

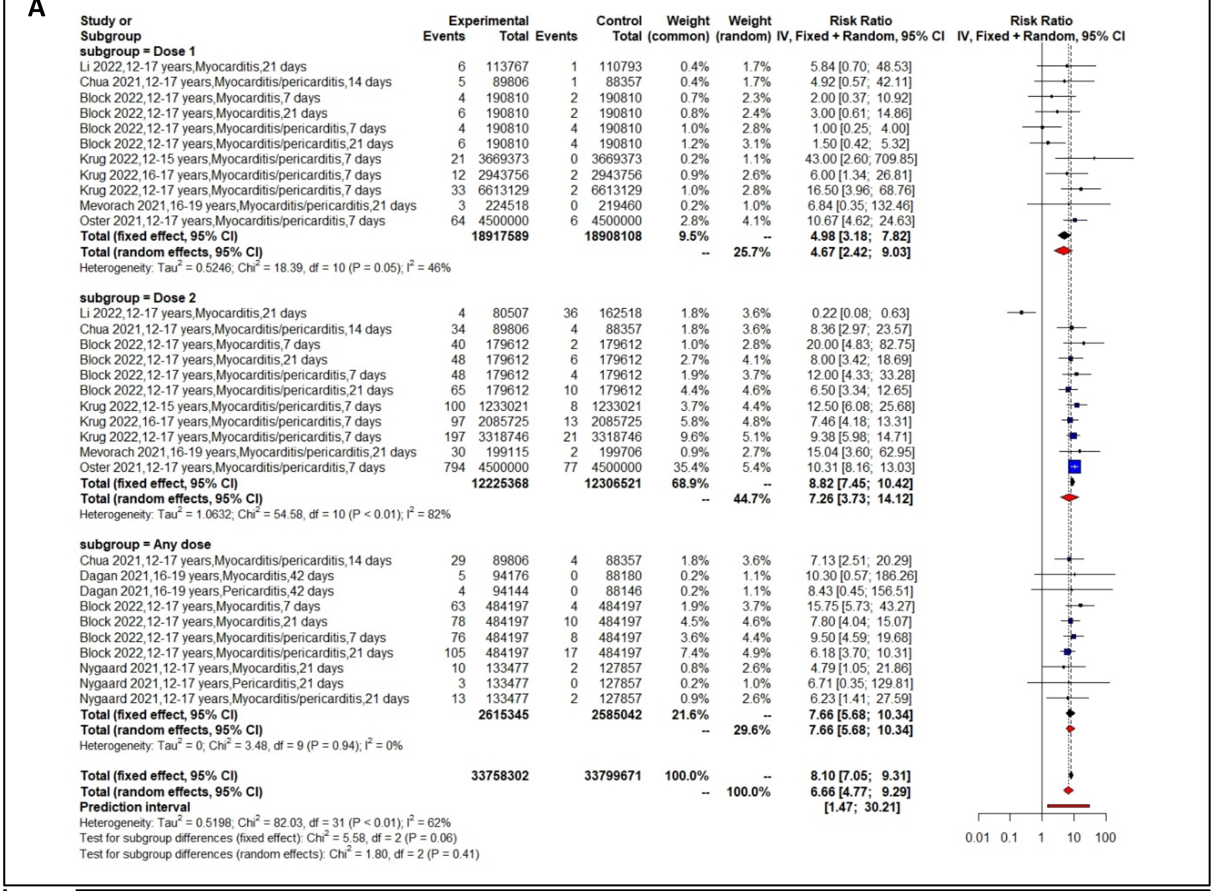

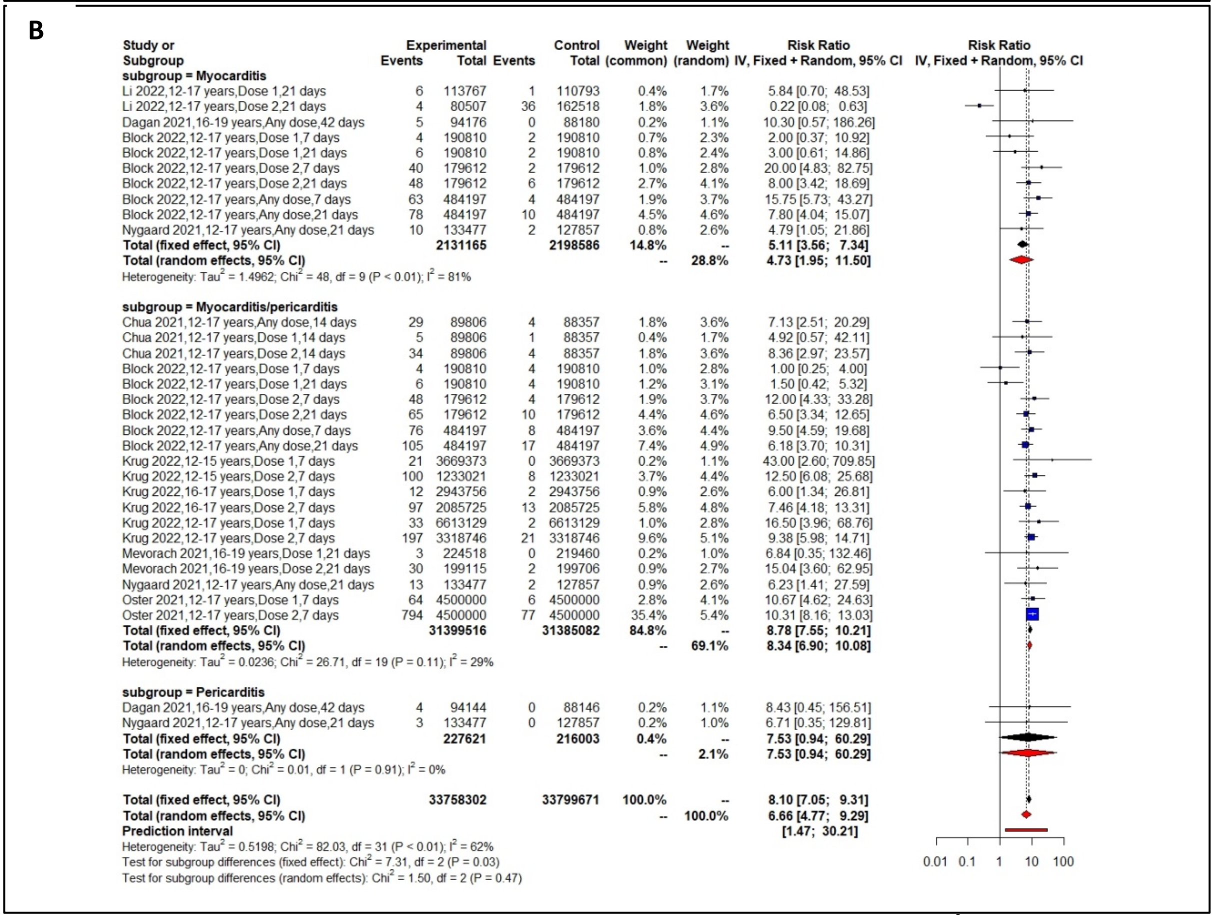

Supplementary Figure 8: **Comparing rates of myocarditis or of myocarditis/pericarditis by gender after BNT162b2 vaccination against COVID-19 among adolescents**. In **panel A**, subgroups are by type of dose while in **panel B**, subgroups consider type of inflammatory heart conditions. In both panels A and B, experimental refers to males and control to females. The whisker represents the 95% confidence interval. The inclusion period of participants spanned from December 2020 to February 2022. Days indicate the period length after BNT162b2 vaccination used by the study. E.g.: 7 days will read as 0-7 days post BNT162b2

Study or Subgroup	Events	Total	Weight	Events per 100000 observations IV, Random, 95% CI
**subgroup = All**				
Li 2022,12-17 years,Myocarditis,Dose 1,21 days	7	224560	1.3%	3.12 [1.25; 6.42]
Li 2022,12-17 years,Myocarditis,Dose 2,21 days	32	82011	1.2%	39.02 [26.69; 55.08]
Choe 2021,16-18 years,Myocarditis/pericarditis,Dose 1,14 days	8	444313	1.3%	1.80 [0.78; 3.55]
Choe 2021,16-18 years,Myocarditis/pericarditis,Dose 2,14 days	19	442025	1.3%	4.30 [2.59; 6.71]
Chua 2021,12-17 years,Myocarditis/pericarditis,Any dose,14 days	33	178163	1.3%	18.52 [12.75; 26.01]
Chua 2021,12-17 years,Myocarditis/pericarditis,Dose 1,14 days	6	178163	1.3%	3.37 [1.24; 7.33]
Chua 2021,12-17 years,Myocarditis/pericarditis,Dose 2,14 days	38	178163	1.3%	21.33 [15.09; 29.27]
Hause 2021,12-17 years,Myocarditis,Any dose,7 days	397	8900000	1.3%	4.46 [4.03; 4.92]
Hause 2022,12-17 years,Myocarditis,Booster dose,7 days	32	2800000	1.3%	1.14 [0.78; 1.61]
Lai 2021,12-18 years,Myocarditis,Dose 1,28 days	8	274884	1.3%	2.91 [1.26; 5.73]
Lai 2021,12-18 years,Myocarditis,Dose 2,28 days	30	237964	1.3%	12.61 [8.51; 18.00]
Nygaard 2021,12-17 years,Myocarditis,Any dose,21 days	12	261334	1.3%	4.59 [2.37; 8.02]
Nygaard 2021,12-17 years,Pericarditis,Any dose,21 days	3	261334	1.3%	1.15 [0.24; 3.35]
Nygaard 2021,12-17 years,Myocarditis/pericarditis,Any dose,21 days	15	261334	1.3%	5.74 [3.21; 9.47]
**Total (95% CI)**		**14724248**	**18.1%**	**6.51 [3.17; 11.05]**
Heterogeneity: Tau^2 = < 0.0001; Chi^2 = 289.79, df = 13 (P < 0.01); I^2 = 96%				
**subgroup = Male**				
Li 2022,12-17 years,Myocarditis,Dose 1,21 days	6	113767	1.2%	5.27 [1.94; 11.48]
Li 2022,12-17 years,Myocarditis,Dose 2,21 days	4	80507	1.2%	4.97 [1.35; 12.72]
Chua 2021,12-17 years,Myocarditis/pericarditis,Any dose,14 days	29	89806	1.2%	32.29 [21.63; 46.37]
Chua 2021,12-17 years,Myocarditis/pericarditis,Dose 1,14 days	5	89806	1.2%	5.57 [1.81; 12.99]
Chua 2021,12-17 years,Myocarditis/pericarditis,Dose 2,14 days	34	89806	1.2%	37.86 [26.22; 52.90]
Dagan 2021,16-19 years,Myocarditis,Any dose,42 days	5	94176	1.2%	5.31 [1.72; 12.39]
Dagan 2021,16-19 years,Pericarditis,Any dose,42 days	4	94144	1.2%	4.25 [1.16; 10.88]
Block 2022,12-17 years,Myocarditis,Dose 1,7 days	4	190810	1.3%	2.10 [0.57; 5.37]
Block 2022,12-17 years,Myocarditis,Dose 1,21 days	6	190810	1.3%	3.14 [1.15; 6.84]
Block 2022,12-17 years,Myocarditis/pericarditis,Dose 1,7 days	4	190810	1.3%	2.10 [0.57; 5.37]
Block 2022,12-17 years,Myocarditis/pericarditis,Dose 1,21 days	6	190810	1.3%	3.14 [1.15; 6.84]
Block 2022,12-17 years,Myocarditis,Dose 2,7 days	40	179612	1.3%	22.27 [15.91; 30.32]
Block 2022,12-17 years,Myocarditis,Dose 2,21 days	48	179612	1.3%	26.72 [19.71; 35.43]
Block 2022,12-17 years,Myocarditis/pericarditis,Dose 2,7 days	48	179612	1.3%	26.72 [19.71; 35.43]
Block 2022,12-17 years,Myocarditis/pericarditis,Dose 2,21 days	65	179612	1.3%	36.19 [27.93; 46.12]
Block 2022,12-17 years,Myocarditis,Any dose,7 days	63	484197	1.3%	13.01 [10.00; 16.65]
Block 2022,12-17 years,Myocarditis,Any dose,21 days	78	484197	1.3%	16.11 [12.73; 20.10]
Block 2022,12-17 years,Myocarditis/pericarditis,Any dose,7 days	76	484197	1.3%	15.70 [12.37; 19.65]
Block 2022,12-17 years,Myocarditis/pericarditis,Any dose,21 days	105	484197	1.3%	21.69 [17.74; 26.25]
Krug 2022,12-15 years,Myocarditis/pericarditis,Dose 1,7 days	21	3669373	1.3%	0.57 [0.35; 0.87]
Krug 2022,12-15 years,Myocarditis/pericarditis,Dose 2,7 days	100	1233021	1.3%	8.11 [6.60; 9.86]
Krug 2022,16-17 years,Myocarditis/pericarditis,Dose 1,7 days	12	2943756	1.3%	0.41 [0.21; 0.71]
Krug 2022,16-17 years,Myocarditis/pericarditis,Dose 2,7 days	97	2085725	1.3%	4.65 [3.77; 5.67]
Krug 2022,12-17 years,Myocarditis/pericarditis,Dose 1,7 days	33	6613129	1.3%	0.50 [0.34; 0.70]
Krug 2022,12-17 years,Myocarditis/pericarditis,Dose 2,7 days	197	3318746	1.3%	5.94 [5.14; 6.83]
Mevorach 2021,16-19 years,Myocarditis/pericarditis,Dose 1,21 days	3	224518	1.3%	1.34 [0.28; 3.90]
Mevorach 2021,16-19 years,Myocarditis/pericarditis,Dose 2,21 days	30	199115	1.3%	15.07 [10.17; 21.51]
Nygaard 2021,12-17 years,Myocarditis,Any dose,21 days	10	133477	1.2%	7.49 [3.59; 13.78]
Nygaard 2021,12-17 years,Pericarditis,Any dose,21 days	3	133477	1.2%	2.25 [0.46; 6.57]
Nygaard 2021,12-17 years,Myocarditis/pericarditis,Any dose,21 days	13	133477	1.2%	9.74 [5.19; 16.65]
Oster 2021,12-17 years,Myocarditis/pericarditis,Dose 1,7 days	64	4500000	1.3%	1.42 [1.10; 1.82]
Oster 2021,12-17 years,Myocarditis/pericarditis,Dose 2,7 days	794	4500000	1.3%	17.64 [16.44; 18.92]
**Total (95% CI)**		**33758302**	**40.9%**	**8.58 [5.60; 12.20]**
Heterogeneity: Tau^2 = < 0.0001; Chi^2 = 2755.26, df = 31 (P = 0); I^2 = 99%				
**subgroup = Female**				
Li 2022,12-17 years,Myocarditis,Dose 1,21 days	1	110793	1.2%	0.90 [0.02; 5.03]
Li 2022,12-17 years,Myocarditis,Dose 2,21 days	36	162518	1.3%	22.15 [15.52; 30.67]
Chua 2021,12-17 years,Myocarditis/pericarditis,Any dose,14 days	4	88357	1.2%	4.53 [1.23; 11.59]
Chua 2021,12-17 years,Myocarditis/pericarditis,Dose 1,14 days	1	88357	1.2%	1.13 [0.03; 6.31]
Chua 2021,12-17 years,Myocarditis/pericarditis,Dose 2,14 days	4	88357	1.2%	4.53 [1.23; 11.59]
Dagan 2021,16-19 years,Myocarditis,Any dose,42 days	0	88180	1.2%	0.00 [0.00; 4.18]
Dagan 2021,16-19 years,Pericarditis,Any dose,42 days	0	88146	1.2%	0.00 [0.00; 4.18]
Block 2022,12-17 years,Myocarditis,Dose 1,7 days	2	190810	1.3%	1.05 [0.13; 3.79]
Block 2022,12-17 years,Myocarditis,Dose 1,21 days	2	190810	1.3%	1.05 [0.13; 3.79]
Block 2022,12-17 years,Myocarditis/pericarditis,Dose 1,7 days	4	190810	1.3%	2.10 [0.57; 5.37]
Block 2022,12-17 years,Myocarditis/pericarditis,Dose 1,21 days	4	190810	1.3%	2.10 [0.57; 5.37]
Block 2022,12-17 years,Myocarditis,Dose 2,7 days	2	179612	1.3%	1.11 [0.13; 4.02]
Block 2022,12-17 years,Myocarditis,Dose 2,21 days	6	179612	1.3%	3.34 [1.23; 7.27]
Block 2022,12-17 years,Myocarditis/pericarditis,Dose 2,7 days	4	179612	1.3%	2.23 [0.61; 5.70]
Block 2022,12-17 years,Myocarditis/pericarditis,Dose 2,21 days	10	179612	1.3%	5.57 [2.67; 10.24]
Block 2022,12-17 years,Myocarditis,Any dose,7 days	4	484197	1.3%	0.83 [0.23; 2.12]
Block 2022,12-17 years,Myocarditis,Any dose,21 days	10	484197	1.3%	2.07 [0.99; 3.80]
Block 2022,12-17 years,Myocarditis/pericarditis,Any dose,7 days	8	484197	1.3%	1.65 [0.71; 3.26]
Block 2022,12-17 years,Myocarditis/pericarditis,Any dose,21 days	17	484197	1.3%	3.51 [2.05; 5.62]
Krug 2022,12-15 years,Myocarditis/pericarditis,Dose 1,7 days	0	3669373	1.3%	0.00 [0.00; 0.10]
Krug 2022,12-15 years,Myocarditis/pericarditis,Dose 2,7 days	8	1233021	1.3%	0.65 [0.28; 1.28]
Krug 2022,16-17 years,Myocarditis/pericarditis,Dose 1,7 days	2	2943756	1.3%	0.07 [0.01; 0.25]
Krug 2022,16-17 years,Myocarditis/pericarditis,Dose 2,7 days	13	2085725	1.3%	0.62 [0.33; 1.07]
Krug 2022,12-17 years,Myocarditis/pericarditis,Dose 1,7 days	2	6613129	1.3%	0.03 [0.00; 0.11]
Krug 2022,12-17 years,Myocarditis/pericarditis,Dose 2,7 days	21	3318746	1.3%	0.63 [0.39; 0.97]
Mevorach 2021,16-19 years,Myocarditis/pericarditis,Dose 1,21 days	0	219460	1.3%	0.00 [0.00; 1.68]
Mevorach 2021,16-19 years,Myocarditis/pericarditis,Dose 2,21 days	2	199706	1.3%	1.00 [0.12; 3.62]
Nygaard 2021,12-17 years,Myocarditis,Any dose,21 days	2	127857	1.2%	1.56 [0.19; 5.65]
Nygaard 2021,12-17 years,Pericarditis,Any dose,21 days	0	127857	1.2%	0.00 [0.00; 2.89]
Nygaard 2021,12-17 years,Myocarditis/pericarditis,Any dose,21 days	2	127857	1.2%	1.56 [0.19; 5.65]
Oster 2021,12-17 years,Myocarditis/pericarditis,Dose 1,7 days	6	4500000	1.3%	0.13 [0.05; 0.29]
Oster 2021,12-17 years,Myocarditis/pericarditis,Dose 2,7 days	77	4500000	1.3%	1.71 [1.35; 2.14]
**Total (95% CI)**		**33799671**	**41.0%**	**1.23 [0.63; 2.02]**
Heterogeneity: Tau^2 = < 0.0001; Chi^2 = 473.79, df = 31 (P < 0.01); I^2 = 93%				
**Total (95% CI)**		**82282221**	**100.0%**	**4.50 [3.14; 6.11]**
**Prediction interval**				**[0.00; 27.12]**
Heterogeneity: Tau^2 < 0.0001; Chi^2 = 4947.07, df = 77 (P = 0); I^2 = 98%				
Test for subgroup differences: Chi^2 = 36.45, df = 2 (P < 0.01)				

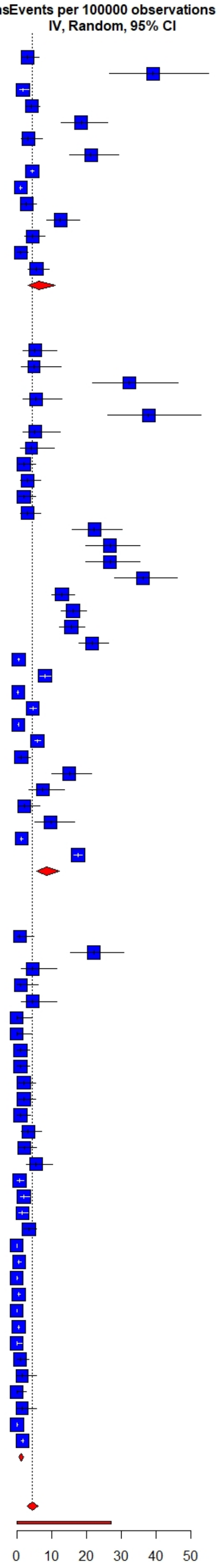

Supplementary Figure 9: Pooled proportion of myocarditis or pericarditis or of myocarditis/pericarditis stratified by sex after BNT162b2 vaccination against COVID-19 among adolescents. The whisker represents the 95% confidence interval. The inclusion period of participants spanned from December 2020 to February 2022

Study or Subgroup	Experimental Events	Experimental Total	Control Events	Control Total	Weight (common)	Weight (random)	Risk Ratio IV, Fixed + Random, 95% CI
**subgroup = 12-15 years**							
Krug 2022,Myocarditis/pericarditis,Dose 1,7 days	21	3669373	0	3669373	0.2%	1.1%	43.00 [2.60; 709.85]
Krug 2022,Myocarditis/pericarditis,Dose 2,7 days	100	1233021	8	1233021	3.7%	4.4%	12.50 [6.08; 25.68]
**Total (fixed effect, 95% CI)**		**4902394**		**4902394**	**4.0%**	--	**13.49 [6.72; 27.10]**
**Total (random effects, 95% CI)**					--	**5.5%**	**13.49 [6.72; 27.10]**
Heterogeneity: $Tau^2 = 0$; $Chi^2 = 0.7$, df = 1 (P = 0.40); $I^2 = 0\%$							
**subgroup = 12-17 years**							
Li 2022,Myocarditis,Dose 1,21 days	6	113767	1	110793	0.4%	1.7%	5.84 [0.70; 48.53]
Li 2022,Myocarditis,Dose 2,21 days	4	80507	36	162518	1.8%	3.6%	0.22 [0.08; 0.63]
Chua 2021,Myocarditis/pericarditis,Any dose,14 days	29	89806	4	88357	1.8%	3.6%	7.13 [2.51; 20.29]
Chua 2021,Myocarditis/pericarditis,Dose 1,14 days	5	89806	1	88357	0.4%	1.7%	4.92 [0.57; 42.11]
Chua 2021,Myocarditis/pericarditis,Dose 2,14 days	34	89806	4	88357	1.8%	3.6%	8.36 [2.97; 23.57]
Block 2022,Myocarditis,Dose 1,7 days	4	190810	2	190810	0.7%	2.3%	2.00 [0.37; 10.92]
Block 2022,Myocarditis,Dose 1,21 days	6	190810	2	190810	0.8%	2.4%	3.00 [0.61; 14.86]
Block 2022,Myocarditis/pericarditis,Dose 1,7 days	4	190810	4	190810	1.0%	2.8%	1.00 [0.25; 4.00]
Block 2022,Myocarditis/pericarditis,Dose 1,21 days	6	190810	4	190810	1.2%	3.1%	1.50 [0.42; 5.32]
Block 2022,Myocarditis,Dose 2,7 days	40	179612	2	179612	1.0%	2.8%	20.00 [4.83; 82.75]
Block 2022,Myocarditis,Dose 2,21 days	48	179612	6	179612	2.7%	4.1%	8.00 [3.42; 18.69]
Block 2022,Myocarditis/pericarditis,Dose 2,7 days	48	179612	4	179612	1.9%	3.7%	12.00 [4.33; 33.28]
Block 2022,Myocarditis/pericarditis,Dose 2,21 days	65	179612	10	179612	4.4%	4.6%	6.50 [3.34; 12.65]
Block 2022,Myocarditis,Any dose,7 days	63	484197	4	484197	1.9%	3.7%	15.75 [5.73; 43.27]
Block 2022,Myocarditis,Any dose,21 days	78	484197	10	484197	4.5%	4.6%	7.80 [4.04; 15.07]
Block 2022,Myocarditis/pericarditis,Any dose,7 days	76	484197	8	484197	3.6%	4.4%	9.50 [4.59; 19.68]
Block 2022,Myocarditis/pericarditis,Any dose,21 days	105	484197	17	484197	7.4%	4.9%	6.18 [3.70; 10.31]
Krug 2022,Myocarditis/pericarditis,Dose 1,7 days	33	6613129	2	6613129	1.0%	2.8%	16.50 [3.96; 68.76]
Krug 2022,Myocarditis/pericarditis,Dose 2,7 days	197	3318746	21	3318746	9.6%	5.1%	9.38 [5.98; 14.71]
Nygaard 2021,Myocarditis,Any dose,21 days	10	133477	2	127857	0.8%	2.6%	4.79 [1.05; 21.86]
Nygaard 2021,Pericarditis,Any dose,21 days	3	133477	0	127857	0.2%	1.0%	6.71 [0.35; 129.81]
Nygaard 2021,Myocarditis/pericarditis,Any dose,21 days	13	133477	2	127857	0.9%	2.6%	6.23 [1.41; 27.59]
Oster 2021,Myocarditis/pericarditis,Dose 1,7 days	64	4500000	6	4500000	2.8%	4.1%	10.67 [4.62; 24.63]
Oster 2021,Myocarditis/pericarditis,Dose 2,7 days	794	4500000	77	4500000	35.4%	5.4%	10.31 [8.16; 13.03]
**Total (fixed effect, 95% CI)**		**23214474**		**23272304**	**87.8%**	--	**7.92 [6.83; 9.19]**
**Total (random effects, 95% CI)**					--	**81.2%**	**5.95 [3.97; 8.92]**
Heterogeneity: $Tau^2 = 0.6694$; $Chi^2 = 78.2$, df = 23 (P < 0.01); $I^2 = 71\%$							
**subgroup = 16-17 years**							
Krug 2022,Myocarditis/pericarditis,Dose 1,7 days	12	2943756	2	2943756	0.9%	2.6%	6.00 [1.34; 26.81]
Krug 2022,Myocarditis/pericarditis,Dose 2,7 days	97	2085725	13	2085725	5.8%	4.8%	7.46 [4.18; 13.31]
**Total (fixed effect, 95% CI)**		**5029481**		**5029481**	**6.6%**	--	**7.25 [4.23; 12.44]**
**Total (random effects, 95% CI)**					--	**7.4%**	**7.25 [4.23; 12.44]**
Heterogeneity: $Tau^2 = 0$; $Chi^2 = 0.07$, df = 1 (P = 0.79); $I^2 = 0\%$							
**subgroup = 16-19 years**							
Dagan 2021,Myocarditis,Any dose,42 days	5	94176	0	88180	0.2%	1.1%	10.30 [0.57; 186.26]
Dagan 2021,Pericarditis,Any dose,42 days	4	94144	0	88146	0.2%	1.1%	8.43 [0.45; 156.51]
Mevorach 2021,Myocarditis/pericarditis,Dose 1,21 days	3	224518	0	219460	0.2%	1.0%	6.84 [0.35; 132.46]
Mevorach 2021,Myocarditis/pericarditis,Dose 2,21 days	30	199115	2	199706	0.9%	2.7%	15.04 [3.60; 62.95]
**Total (fixed effect, 95% CI)**		**611953**		**595492**	**1.6%**	--	**11.81 [3.96; 35.21]**
**Total (random effects, 95% CI)**					--	**5.9%**	**11.81 [3.96; 35.21]**
Heterogeneity: $Tau^2 = 0$; $Chi^2 = 0.3$, df = 3 (P = 0.96); $I^2 = 0\%$							
**Total (fixed effect, 95% CI)**		**33758302**		**33799671**	**100.0%**	--	**8.10 [7.05; 9.31]**
**Total (random effects, 95% CI)**					--	**100.0%**	**6.66 [4.77; 9.29]**
**Prediction interval**							**[1.47; 30.21]**
Heterogeneity: $Tau^2 = 0.5198$; $Chi^2 = 82.03$, df = 31 (P < 0.01); $I^2 = 62\%$							
Test for subgroup differences (fixed effect): $Chi^2 = 2.76$, df = 3 (P = 0.43)							
Test for subgroup differences (random effects): $Chi^2 = 4.66$, df = 3 (P = 0.20)							

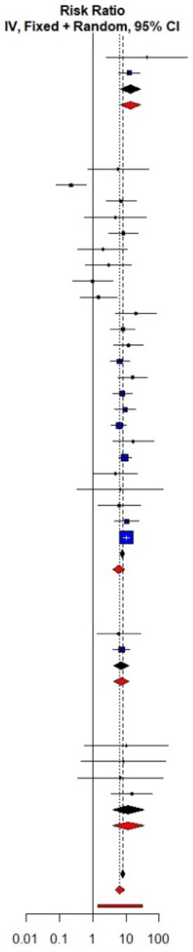

Supplementary Figure 10: **Comparing rates of myocarditis or of myocarditis/pericarditis by gender after BNT162b2 vaccination against COVID-19 among adolescents.** Subgroups are by age-groups. Experimental refers to males and control to females. The whisker represents the 95% confidence interval. The inclusion period of participants spanned from December 2020 to February 2022.

Study or Subgroup	Events	Total	Weight	Events per 100000 observations IV, Random, 95% CI
**subgroup = >7 days**				
Choe 2021,16-18 years,Myocarditis/pericarditis (All),Dose 1	8	444313	1.3%	1.80 [0.78; 3.55]
Choe 2021,16-18 years,Myocarditis/pericarditis (All),Dose 2	19	442025	1.3%	4.30 [2.59; 6.71]
Chua 2021,12-17 years,Myocarditis/pericarditis (All),Any dose	33	178163	1.3%	18.52 [12.75; 26.01]
Chua 2021,12-17 years,Myocarditis/pericarditis (Males),Any dose	29	89806	1.2%	32.29 [21.63; 46.37]
Chua 2021,12-17 years,Myocarditis/pericarditis (Females),Any dose	4	88357	1.2%	4.53 [1.23; 11.59]
Chua 2021,12-17 years,Myocarditis/pericarditis (All),Dose 1	6	178163	1.3%	3.37 [1.24; 7.33]
Chua 2021,12-17 years,Myocarditis/pericarditis (Males),Dose 1	5	89806	1.2%	5.57 [1.81; 12.99]
Chua 2021,12-17 years,Myocarditis/pericarditis (Females),Dose 1	1	88357	1.2%	1.13 [0.03; 6.31]
Chua 2021,12-17 years,Myocarditis/pericarditis (All),Dose 2	38	178163	1.3%	21.33 [15.09; 29.27]
Chua 2021,12-17 years,Myocarditis/pericarditis (Males),Dose 2	34	89806	1.2%	37.86 [26.22; 52.90]
Chua 2021,12-17 years,Myocarditis/pericarditis (Females),Dose 2	4	88357	1.2%	4.53 [1.23; 11.59]
Li 2022,12-17 years,Myocarditis (All),Dose 1	7	224560	1.3%	3.12 [1.25; 6.42]
Li 2022,12-17 years,Myocarditis (Males),Dose 1	6	113767	1.2%	5.27 [1.94; 11.48]
Li 2022,12-17 years,Myocarditis (Females),Dose 1	1	110793	1.2%	0.90 [0.02; 5.03]
Li 2022,12-17 years,Myocarditis (All),Dose 2	32	82011	1.2%	39.02 [26.69; 55.08]
Li 2022,12-17 years,Myocarditis (Males),Dose 2	4	80507	1.2%	4.97 [1.35; 12.72]
Li 2022,12-17 years,Myocarditis (Females),Dose 2	36	162518	1.3%	22.15 [15.52; 30.67]
Block 2022,12-17 years,Myocarditis (Males),Dose 1	6	190810	1.3%	3.14 [1.15; 6.84]
Block 2022,12-17 years,Myocarditis (Females),Dose 1	2	190810	1.3%	1.05 [0.13; 3.79]
Block 2022,12-17 years,Myocarditis/pericarditis (Males),Dose 1	6	190810	1.3%	3.14 [1.15; 6.84]
Block 2022,12-17 years,Myocarditis/pericarditis (Females),Dose 1	4	190810	1.3%	2.10 [0.57; 5.37]
Block 2022,12-17 years,Myocarditis (Males),Dose 2	48	179612	1.3%	26.72 [19.71; 35.43]
Block 2022,12-17 years,Myocarditis (Females),Dose 2	6	179612	1.3%	3.34 [1.23; 7.27]
Block 2022,12-17 years,Myocarditis/pericarditis (Males),Dose 2	65	179612	1.3%	36.19 [27.93; 46.12]
Block 2022,12-17 years,Myocarditis/pericarditis (Females),Dose 2	10	179612	1.3%	5.57 [2.67; 10.24]
Block 2022,12-17 years,Myocarditis (Males),Any dose	78	484197	1.3%	16.11 [12.73; 20.10]
Block 2022,12-17 years,Myocarditis (Females),Any dose	10	484197	1.3%	2.07 [0.99; 3.80]
Block 2022,12-17 years,Myocarditis/pericarditis (Males),Any dose	105	484197	1.3%	21.69 [17.74; 26.25]
Block 2022,12-17 years,Myocarditis/pericarditis (Females),Any dose	17	484197	1.3%	3.51 [2.05; 5.62]
Mevorach 2021,16-19 years,Myocarditis/pericarditis (Males),Dose 1	3	224518	1.3%	1.34 [0.28; 3.90]
Mevorach 2021,16-19 years,Myocarditis/pericarditis (Females),Dose 1	0	219460	1.3%	0.00 [0.00; 1.68]
Mevorach 2021,16-19 years,Myocarditis/pericarditis (Males),Dose 2	30	199115	1.3%	15.07 [10.17; 21.51]
Mevorach 2021,16-19 years,Myocarditis/pericarditis (Females),Dose 2	2	199706	1.3%	1.00 [0.12; 3.62]
Nygaard 2021,12-17 years,Myocarditis (All),Any dose	12	261334	1.3%	4.59 [2.37; 8.02]
Nygaard 2021,12-17 years,Myocarditis (Males),Any dose	10	133477	1.2%	7.49 [3.59; 13.78]
Nygaard 2021,12-17 years,Myocarditis (Females),Any dose	2	127857	1.2%	1.56 [0.19; 5.65]
Nygaard 2021,12-17 years,Pericarditis (All),Any dose	3	261334	1.3%	1.15 [0.24; 3.35]
Nygaard 2021,12-17 years,Pericarditis (Males),Any dose	3	133477	1.2%	2.25 [0.46; 6.57]
Nygaard 2021,12-17 years,Pericarditis (Females),Any dose	0	127857	1.2%	0.00 [0.00; 2.89]
Nygaard 2021,12-17 years,Myocarditis/pericarditis (All),Any dose	15	261334	1.3%	5.74 [3.21; 9.47]
Nygaard 2021,12-17 years,Myocarditis/pericarditis (Males),Any dose	13	133477	1.2%	9.74 [5.19; 16.65]
Nygaard 2021,12-17 years,Myocarditis/pericarditis (Females),Any dose	2	127857	1.2%	1.56 [0.19; 5.65]
Lai 2021,12-18 years,Myocarditis (All),Dose 1	8	274884	1.3%	2.91 [1.26; 5.73]
Lai 2021,12-18 years,Myocarditis (All),Dose 2	30	237964	1.3%	12.61 [8.51; 18.00]
Dagan 2021,16-19 years,Myocarditis (Females),Any dose	0	88180	1.2%	0.00 [0.00; 4.18]
Dagan 2021,16-19 years,Myocarditis (Males),Any dose	5	94176	1.2%	5.31 [1.72; 12.39]
Dagan 2021,16-19 years,Pericarditis (Females),Any dose	0	88146	1.2%	0.00 [0.00; 4.18]
Dagan 2021,16-19 years,Pericarditis (Males),Any dose	4	94144	1.2%	4.25 [1.16; 10.88]
**Total (95% CI)**		**9436245**	**60.4%**	**5.87 [3.83; 8.34]**
Heterogeneity: Tau^2 = < 0.0001; Chi^2 = 854.81, df = 47 (P < 0.01); I^2 = 95%				
**subgroup = 0-7 days**				
Block 2022,12-17 years,Myocarditis (Males),Dose 1	4	190810	1.3%	2.10 [0.57; 5.37]
Block 2022,12-17 years,Myocarditis (Females),Dose 1	2	190810	1.3%	1.05 [0.13; 3.79]
Block 2022,12-17 years,Myocarditis/pericarditis (Males),Dose 1	4	190810	1.3%	2.10 [0.57; 5.37]
Block 2022,12-17 years,Myocarditis/pericarditis (Females),Dose 1	4	190810	1.3%	2.10 [0.57; 5.37]
Block 2022,12-17 years,Myocarditis (Males),Dose 2	40	179612	1.3%	22.27 [15.91; 30.32]
Block 2022,12-17 years,Myocarditis (Females),Dose 2	2	179612	1.3%	1.11 [0.13; 4.02]
Block 2022,12-17 years,Myocarditis/pericarditis (Males),Dose 2	48	179612	1.3%	26.72 [19.71; 35.43]
Block 2022,12-17 years,Myocarditis/pericarditis (Females),Dose 2	4	179612	1.3%	2.23 [0.61; 5.70]
Block 2022,12-17 years,Myocarditis (Males),Any dose	63	484197	1.3%	13.01 [10.00; 16.65]
Block 2022,12-17 years,Myocarditis (Females),Any dose	4	484197	1.3%	0.83 [0.23; 2.12]
Block 2022,12-17 years,Myocarditis/pericarditis (Males),Any dose	76	484197	1.3%	15.70 [12.37; 19.65]
Block 2022,12-17 years,Myocarditis/pericarditis (Females),Any dose	8	484197	1.3%	1.65 [0.71; 3.26]
Hause 2021,12-17 years,Myocarditis (All),Any dose	397	8900000	1.3%	4.46 [4.03; 4.92]
Hause 2022,12-17 years,Myocarditis (All),Booster dose	32	2800000	1.3%	1.14 [0.78; 1.61]
Krug 2022,12-15 years,Myocarditis/pericarditis (Females),Dose 1	0	3669373	1.3%	0.00 [0.00; 0.10]
Krug 2022,12-15 years,Myocarditis/pericarditis (Females),Dose 2	8	1233021	1.3%	0.65 [0.28; 1.28]
Krug 2022,16-17 years,Myocarditis/pericarditis (Females),Dose 1	2	2943756	1.3%	0.07 [0.01; 0.25]
Krug 2022,16-17 years,Myocarditis/pericarditis (Females),Dose 2	13	2085725	1.3%	0.62 [0.33; 1.07]
Krug 2022,12-17 years,Myocarditis/pericarditis (Females),Dose 1	2	6613129	1.3%	0.03 [0.00; 0.11]
Krug 2022,12-17 years,Myocarditis/pericarditis (Females),Dose 2	21	3318746	1.3%	0.63 [0.39; 0.97]
Krug 2022,12-15 years,Myocarditis/pericarditis (Males),Dose 1	21	3669373	1.3%	0.57 [0.35; 0.87]
Krug 2022,12-15 years,Myocarditis/pericarditis (Males),Dose 2	100	1233021	1.3%	8.11 [6.60; 9.86]
Krug 2022,16-17 years,Myocarditis/pericarditis (Males),Dose 1	12	2943756	1.3%	0.41 [0.21; 0.71]
Krug 2022,16-17 years,Myocarditis/pericarditis (Males),Dose 2	97	2085725	1.3%	4.65 [3.77; 5.67]
Krug 2022,12-17 years,Myocarditis/pericarditis (Males),Dose 1	33	6613129	1.3%	0.50 [0.34; 0.70]
Krug 2022,12-17 years,Myocarditis/pericarditis (Males),Dose 2	197	3318746	1.3%	5.94 [5.14; 6.83]
Oster 2021,12-17 years,Myocarditis/pericarditis (Males),Dose 1	64	4500000	1.3%	1.42 [1.10; 1.82]
Oster 2021,12-17 years,Myocarditis/pericarditis (Females),Dose 1	6	4500000	1.3%	0.13 [0.05; 0.29]
Oster 2021,12-17 years,Myocarditis/pericarditis (Males),Dose 2	794	4500000	1.3%	17.64 [16.44; 18.92]
Oster 2021,12-17 years,Myocarditis/pericarditis (Females),Dose 2	77	4500000	1.3%	1.71 [1.35; 2.14]
**Total (95% CI)**		**72845976**	**39.6%**	**2.76 [1.37; 4.62]**
Heterogeneity: Tau^2 = < 0.0001; Chi^2 = 3668.33, df = 29 (P = 0); I^2 = 99%				
**Total (95% CI)**		**82282221**	**100.0%**	**4.50 [3.14; 6.11]**
**Prediction interval**				**[0.00; 27.12]**
Heterogeneity: Tau^2 < 0.0001; Chi^2 = 4947.07, df = 77 (P = 0); I^2 = 98%				
Test for subgroup differences: Chi^2 = 4.89, df = 1 (P = 0.03)				

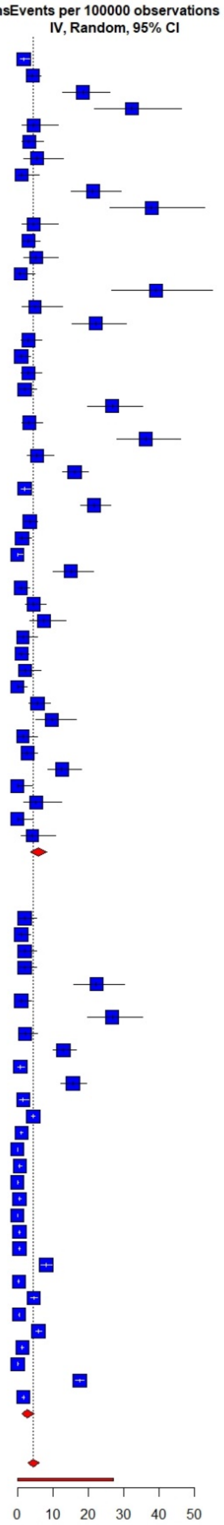

Supplementary Figure 11: Pooled cumulative proportion of myocarditis or pericarditis or of myocarditis/pericarditis before and after seven reporting days of BNT162b2 vaccination against COVID-19 among adolescents. The whisker represents the 95% confidence interval. The inclusion period of participants spanned from December 2020 to February 2022

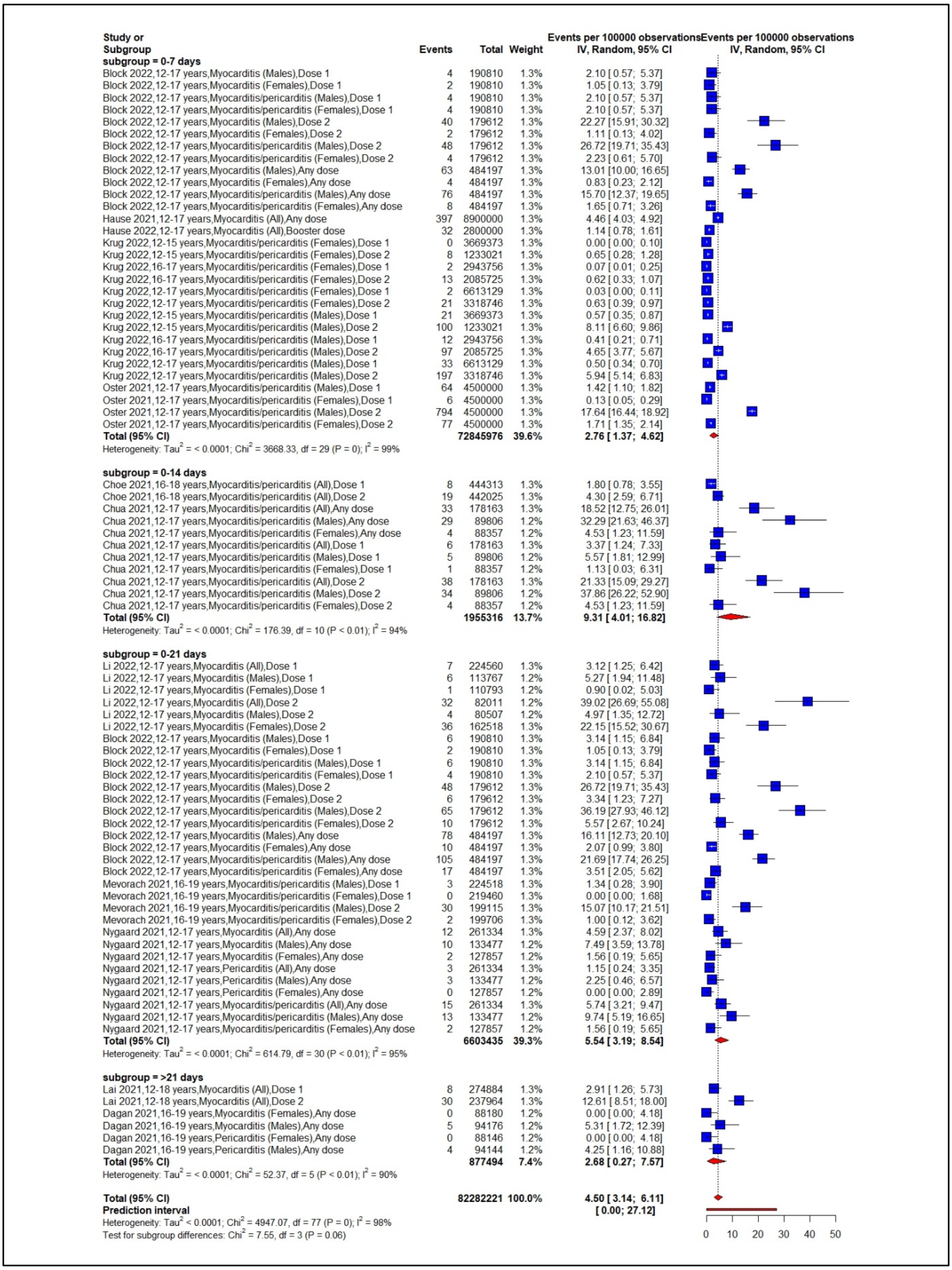

Supplementary Figure 12: **Pooled cumulative proportion of myocarditis or pericarditis or of myocarditis/pericarditis reported after each seven days period across all doses including booster dose of BNT162b2 vaccination against COVID-19 among adolescents**. The whisker represents the 95% confidence interval. The inclusion period of participants spanned from December 2020 to February 2022

A

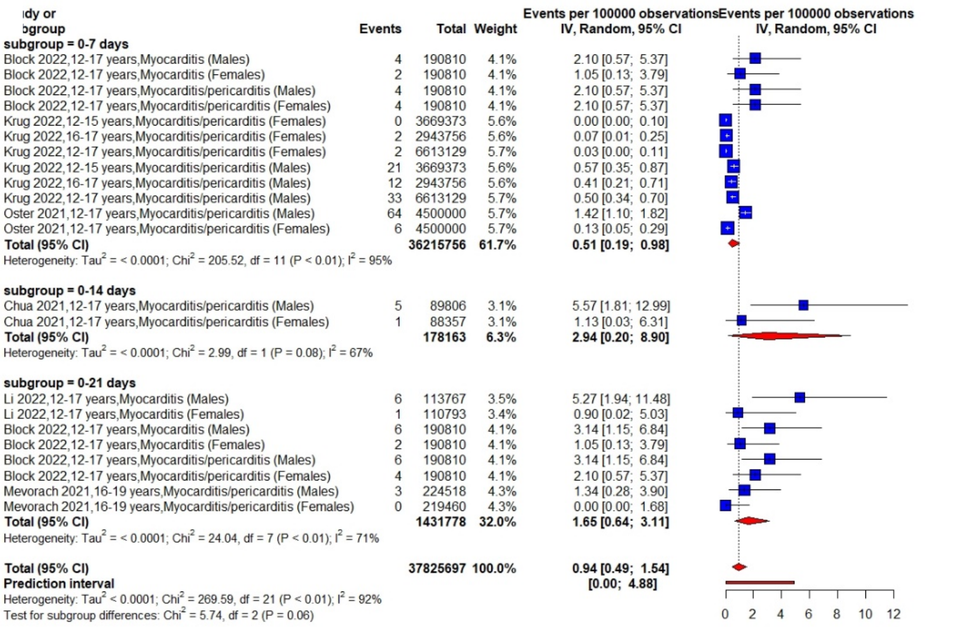

B

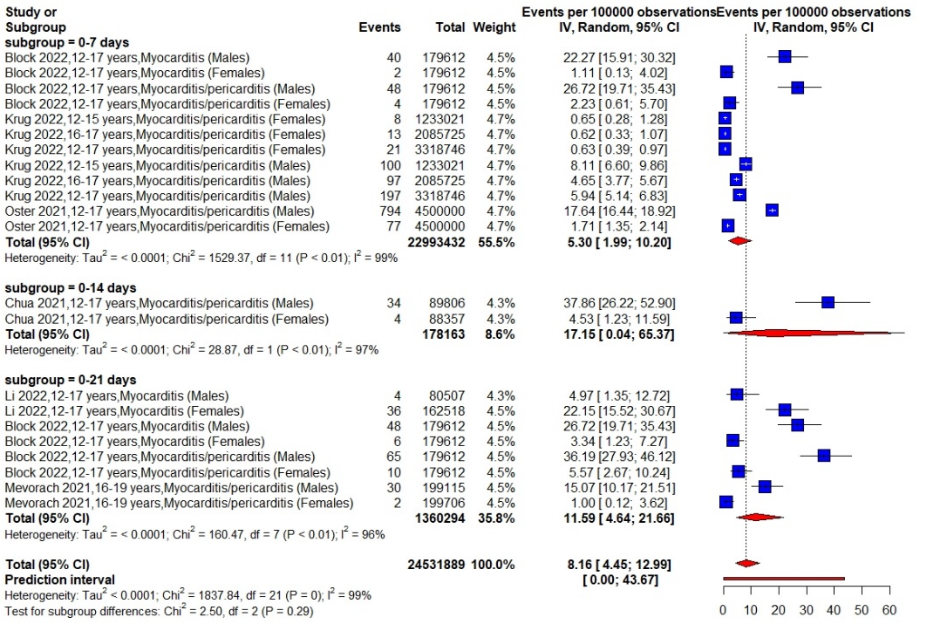

Supplementary Figure 13: Pooled cumulative proportion of myocarditis or pericarditis or of myocarditis/pericarditis reported after each seven days period after the first (Panel A) and the second dose (Panel B) of BNT162b2 vaccination against COVID-19 among adolescents. The whisker represents the 95% confidence interval. The inclusion period of participants spanned from December 2020 to February 2022

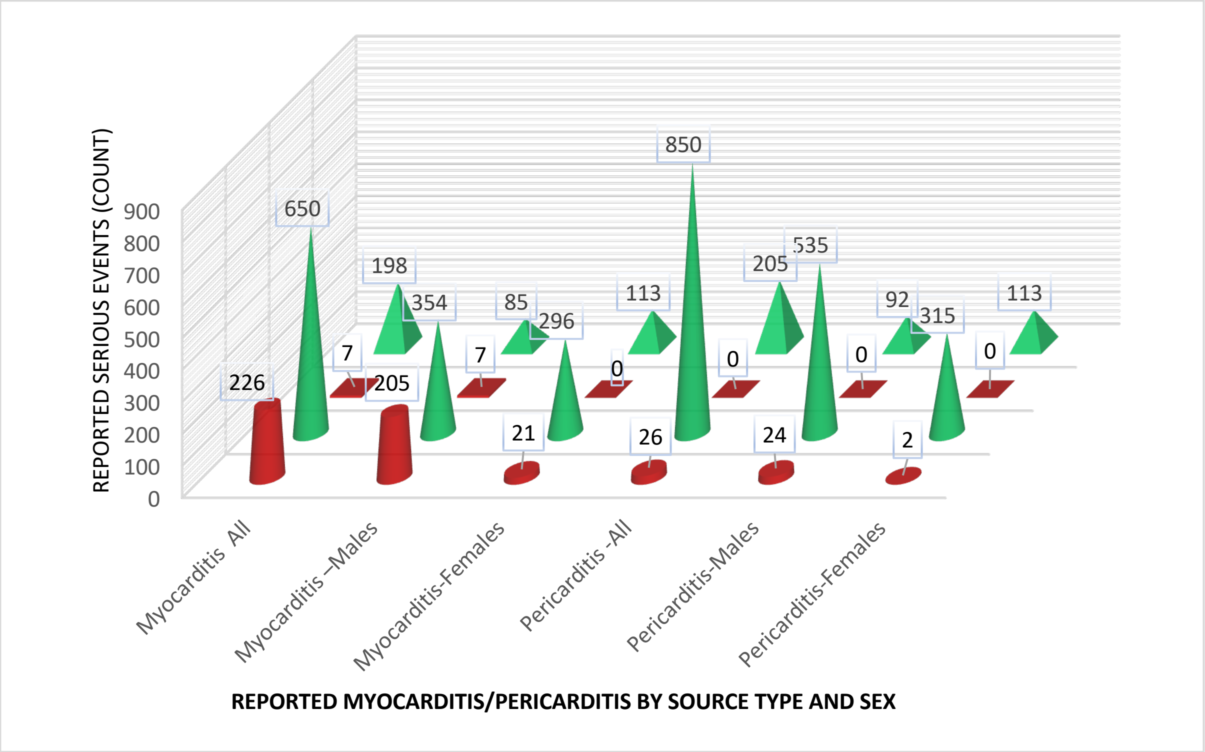

Supplementary Figure 14: Reporting of myopericarditis among adolescents aged 12-17 years using data from the passive surveillance, nested in the World Health Organisation database (VigiBase) of spontaneous reports (adapted from Chouchana et al 2022). Partial cones (red) represent reporting number of myopericarditis in mRNA COVID-19 vaccine recipients (12-17 years) according to patient sex within the WHO global safety database compared to reported serious reports (green full cones). Partial pyramids (red) represent reporting number of myopericarditis in mRNA COVID-19 vaccine recipients (12-17 years) according to patient sex within the WHO global safety database compared to serious report originating from healthcare professionals only (full pyramids, red).

References

1. Block, J. P. Cardiac Complications After SARS-CoV-2 Infection and mRNA COVID-19 Vaccination — PCORnet, United States, January 2021–January 2022. *MMWR Morb Mortal Wkly Rep* **71**, (2022).
2. Chua, G. T. *et al.* Epidemiology of Acute Myocarditis/Pericarditis in Hong Kong Adolescents Following Comirnaty Vaccination. *Clin Infect Dis* (2021) doi:10.1093/cid/ciab989.
3. Chouchana, L. *et al.* Features of Inflammatory Heart Reactions Following mRNA COVID-19 Vaccination at a Global Level. *Clin Pharmacol Ther* **111**, 605–613 (2022).
4. Karlstad, Ø. *et al.* SARS-CoV-2 Vaccination and Myocarditis in a Nordic Cohort Study of 23 Million Residents. *JAMA Cardiology* **7**, 600–612 (2022).
5. Li, X. *et al.* Myocarditis Following COVID-19 BNT162b2 Vaccination Among Adolescents in Hong Kong. *JAMA Pediatr* (2022) doi:10.1001/jamapediatrics.2022.0101.
6. Funk, P. R. *et al.* Benefit-risk assessment of COVID-19 vaccine, mRNA (Comirnaty) for age 16-29 years. *Vaccine* **40**, 2781–2789 (2022).
7. Krug, A., Stevenson, J. & Høeg, T. B. BNT162b2 Vaccine-Associated Myo/Pericarditis in Adolescents: A Stratified Risk-Benefit Analysis. *European Journal of Clinical Investigation* **n/a**, e13759.
8. Nygaard, U. *et al.* Population-based Incidence of Myopericarditis After COVID-19 Vaccination in Danish Adolescents. *Pediatr Infect Dis J* **41**, e25–e28 (2022).
9. Oster, M. E. *et al.* Myocarditis Cases Reported After mRNA-Based COVID-19 Vaccination in the US From December 2020 to August 2021. *JAMA* **327**, 331–340 (2022).
10. Yap, J. *et al.* Pericarditis and myocarditis after COVID-19 mRNA vaccination in a nationwide setting. *Ann Acad Med Singap* **51**, 96–100 (2022).

11. Das, B. B. *et al.* Myopericarditis after messenger RNA Coronavirus Disease 2019 Vaccination in Adolescents 12 to 18 Years of Age. *J Pediatr* **238**, 26-32.e1 (2021).
12. Foltran, D. *et al.* Myocarditis and pericarditis in adolescents after first and second doses of mRNA COVID-19 vaccines. *Eur Heart J Qual Care Clin Outcomes* **8**, 99–103 (2022).
13. Gurdasani, D. *et al.* Vaccinating adolescents against SARS-CoV-2 in England: a risk-benefit analysis. *J R Soc Med* **114**, 513–524 (2021).
14. Dagan, N., Barda, N. & Balicer, R. D. Adverse Effects after BNT162b2 Vaccine and SARS-CoV-2 Infection, According to Age and Sex. *N Engl J Med* **385**, 2299 (2021).
15. Hause, A. M. COVID-19 Vaccine Safety in Adolescents Aged 12–17 Years — United States, December 14, 2020–July 16, 2021. *MMWR Morb Mortal Wkly Rep* **70**, (2021).
16. Hause, A. M. Safety Monitoring of COVID-19 Vaccine Booster Doses Among Persons Aged 12–17 Years — United States, December 9, 2021–February 20, 2022. *MMWR Morb Mortal Wkly Rep* **71**, (2022).
17. Mevorach, D. *et al.* Myocarditis after BNT162b2 Vaccination in Israeli Adolescents. *N Engl J Med* **386**, 998–999 (2022).
18. Jain, S. S. *et al.* COVID-19 Vaccination-Associated Myocarditis in Adolescents. *Pediatrics* **148**, e2021053427 (2021).
19. June Choe, Y. *et al.* Safety and effectiveness of BNT162b2 mRNA Covid-19 vaccine in adolescents. *Vaccine* **40**, 691–694 (2022).
20. Lai, F. T. T. *et al.* Adverse events of special interest following the use of BNT162b2 in adolescents: a population-based retrospective cohort study. *Emerg Microbes Infect* **11**, 885–893 (2022).
