# Supplementary material for: Systematic review and meta-analysis of myocarditis and pericarditis in adolescents following COVID-19 BNT162b2 vaccination
Source: NPJ Vaccines. 2023 Jun 9;8:89. doi: 10.1038/s41541-023-00681-3 (PMC10252172; doi:10.1038/s41541-023-00681-3)
Supplement: Supplementary file 2 — REPORTING SUMMARY [file 41541_2023_681_MOESM2_ESM.pdf]

Corresponding author(s): Prof. Patrick DMC KATOTO
Last updated by author(s): Jan 29, 2023

Reporting Summary

Nature Portfolio wishes to improve the reproducibility of the work that we publish. This form provides structure for consistency and transparency in reporting. For further information on Nature Portfolio policies, see our Editorial Policies and the Editorial Policy Checklist.

Statistics

For all statistical analyses, confirm that the following items are present in the figure legend, table legend, main text, or Methods section.

n/a	Confirmed	
☐	☒	The exact sample size (*n*) for each experimental group/condition, given as a discrete number and unit of measurement
☒	☐	A statement on whether measurements were taken from distinct samples or whether the same sample was measured repeatedly
☐	☒	The statistical test(s) used AND whether they are one- or two-sided<br>*Only common tests should be described solely by name; describe more complex techniques in the Methods section.*
☐	☒	A description of all covariates tested
☒	☐	A description of any assumptions or corrections, such as tests of normality and adjustment for multiple comparisons
☐	☒	A full description of the statistical parameters including central tendency (e.g. means) or other basic estimates (e.g. regression coefficient) AND variation (e.g. standard deviation) or associated estimates of uncertainty (e.g. confidence intervals)
☐	☒	For null hypothesis testing, the test statistic (e.g. *F*, *t*, *r*) with confidence intervals, effect sizes, degrees of freedom and *P* value noted<br>*Give P values as exact values whenever suitable.*
☒	☐	For Bayesian analysis, information on the choice of priors and Markov chain Monte Carlo settings
☒	☐	For hierarchical and complex designs, identification of the appropriate level for tests and full reporting of outcomes
☐	☒	Estimates of effect sizes (e.g. Cohen's *d*, Pearson's *r*), indicating how they were calculated

Our web collection on statistics for biologists contains articles on many of the points above.

Software and code

Policy information about availability of computer code

Data collection	We used Covidence (Veritas Health Innovation, Melbourne, Australia. Available at www.covidence.org.) for data collection (from study screening, to study inclusion and bias assessment) before being exported to Microsoft excel (v 16.69.1)
Data analysis	We analyzed quantitative data using the meta, metaprop and metabin packages (RevMan5 layout) of the statistical software R (version 4.0.3, R Foundation for Statistical Computing, Vienna, Austria).

For manuscripts utilizing custom algorithms or software that are central to the research but not yet described in published literature, software must be made available to editors and reviewers. We strongly encourage code deposition in a community repository (e.g. GitHub). See the Nature Portfolio guidelines for submitting code & software for further information.

Data

Policy information about availability of data

All manuscripts must include a data availability statement. This statement should provide the following information, where applicable:

- Accession codes, unique identifiers, or web links for publicly available datasets
- A description of any restrictions on data availability
- For clinical datasets or third party data, please ensure that the statement adheres to our policy

All data generated or analysed during this study are included in this published article (and its supplementary information files).

Human research participants

Policy information about studies involving human research participants and Sex and Gender in Research.

Reporting on sex and gender	We used a sex-based analysis to compare the estimates of cardiovascular events (myocarditis/pericarditis) in boys and girls following the first and second doses of BNT162b2 vaccination.
Population characteristics	"see above"
Recruitment	We did not recruit participants
Ethics oversight	We did not seek IRB clearance since we did not collect profoundly personal, sensitive, or confidential information from individuals for this systematic review.

Note that full information on the approval of the study protocol must also be provided in the manuscript.

Field-specific reporting

Please select the one below that is the best fit for your research. If you are not sure, read the appropriate sections before making your selection.

☒ Life sciences ☐ Behavioural & social sciences ☐ Ecological, evolutionary & environmental sciences

For a reference copy of the document with all sections, see nature.com/documents/nr-reporting-summary-flat.pdf

Life sciences study design

All studies must disclose on these points even when the disclosure is negative.

Sample size	No statistical sample size calculation was needed. We ensured that we searched at least three databases as per PRISMA.
Data exclusions	No extracted data from included studies was excluded.
Replication	We did not perform an experimental study. Using Boolean operators, we used MeSH terms related to "BNT162b2 vaccination" AND "adolescents" to ensure replication of our included studies.
Randomization	We did not perform randomization (not required for systematic review).
Blinding	Blinding of participants was not also performed (not required for systematic review).

Reporting for specific materials, systems and methods

We require information from authors about some types of materials, experimental systems and methods used in many studies. Here, indicate whether each material, system or method listed is relevant to your study. If you are not sure if a list item applies to your research, read the appropriate section before selecting a response.

Materials & experimental systems

n/a	Involved in the study	
☒	☐	Antibodies
☒	☐	Eukaryotic cell lines
☒	☐	Palaeontology and archaeology
☒	☐	Animals and other organisms
☒	☐	Clinical data
☒	☐	Dual use research of concern

Methods

n/a	Involved in the study	
☒	☐	ChIP-seq
☒	☐	Flow cytometry
☒	☐	MRI-based neuroimaging
